# Direct and Linker-Exchange Alcohol-Assisted Hydrothermal Synthesis of Imide-Linked Covalent Organic Frameworks

**DOI:** 10.1021/acs.chemmater.1c04051

**Published:** 2022-02-17

**Authors:** Johannes Maschita, Tanmay Banerjee, Bettina V. Lotsch

**Affiliations:** † Max Planck Institute for Solid State Research, Heisenbergstraße 1, 70569 Stuttgart, Germany; ‡ Department of Chemistry, University of Munich (LMU), Butenandtstraße 5-13, 81377 München, Germany; § Department of Chemistry, BITS Pilani, Pilani Campus, Rajasthan − 333031, India; ∥ E-conversion and Center for Nanoscience, Schellingstraße 4, 80799 München, Germany

## Abstract

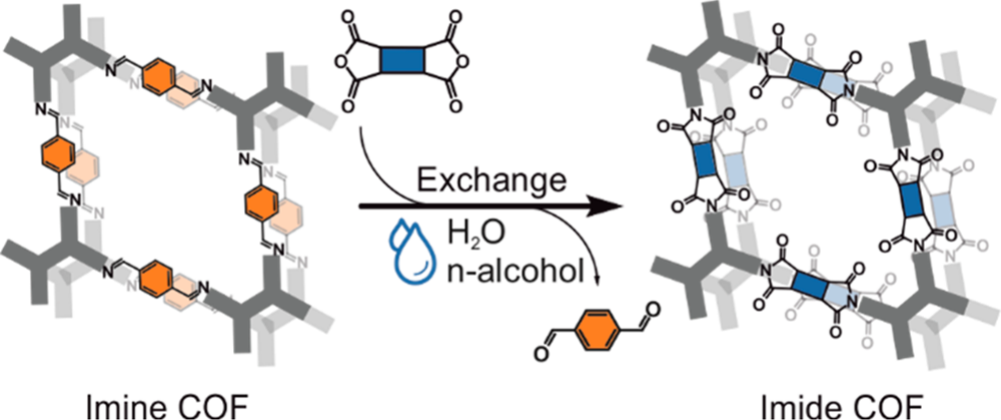

Covalent organic frameworks (COFs) are an extensively studied class of porous materials, which distinguish themselves from other porous polymers in their crystallinity and high degree of modularity, enabling a wide range of applications. However, the established synthetic protocols for the synthesis of stable and crystalline COFs, such as imide-linked COFs, often requires the use of high boiling solvents and toxic catalysts, making their synthesis expensive and environmentally harmful. Herein, we report a new environmentally friendly strategy—an alcohol-assisted hydrothermal polymerization approach (aaHTP) for the synthesis of a wide range of crystalline and porous imide-linked COFs. This method allows us to gain access to new COFs and to avoid toxic solvents by up to 90% through substituting commonly used organic solvent mixtures with water and small amounts of n-alcohols without being restricted to water-soluble linker molecules. Additionally, we use the aaHTP to demonstrate an eco-friendly COF-to-COF transformation of an imine-linked COF into a novel imide-linked COF via linkage replacement, inaccessible using published reaction conditions.

## Introduction

Two-dimensional (2D) covalent organic frameworks (COFs) are built of organic building blocks that form extended planar networks via in-plane covalent bonding and are further stacked in the third dimension by out-of-plane π–π interactions and van-der-Waals forces.^1^

As with many high-performance polymers, for COFs, enhanced crystallinity is a desired feature: not only does it improve mechanical and thermal strength^2,3^ but it can impart directionality to functional properties like electron transport and thus enhance, e.g., electrical conductivity.^4,5^ However, unlike (zero-dimensional) molecules, and one-dimensional (1D) polymers of sufficient molecular flexibility, which may still be recrystallized from solution or a salt-melt, postsynthesis recrystallization of 2D COFs is an outstanding challenge due to the insoluble and infusible nature of COFs, once formed. Therefore, COF synthesis is typically conducted according to the principles of dynamic covalent chemistry: the COF formation reaction has to be reversible to enable crystal defect correction during the polymerization process, carried out under precise thermodynamic and kinetic control.^1,6^ However—bond breaking being as crucial as bond formation in dynamic covalent synthesis—stability is achieved at the expense of crystallinity, frequently leading to poorly crystalline products, rendering synthesis of stable and crystalline COFs challenging.^7^ To achieve sufficient reversibility of the COF formation reaction, toxic and high boiling solvents like mesitylene, 1,2-dioxane, *o*-dichlorobenzene, or *N*-methyl-2-pyrrolidone (NMP) and high temperatures between 120 and 250 °C are usually required.^8^

There is however an increasing need for less harmful synthetic procedures,^9^ and therefore, the development of simple, low cost, and green synthetic protocols for the synthesis of COFs has attracted increasing interest in the past few years, leading to the hydrothermal synthesis of keto-enamine-linked, azine-linked, and imine-linked COFs.^10−12^ The high reversibility of these linkages and the fact that they are commonly produced by adding aqueous acetic acid as a catalyst, aided the development of hydrothermal synthetic protocols for these types of COFs. In contrast, for the synthesis of imide-linked COFs, which are adorned with many beneficial and desired properties, such as high chemical resistance, high thermal stability, and outstanding mechanical properties,^9,13,14^ harsh reaction conditions are required to form these polymers in a crystalline fashion.^13,15^ In fact, there are only two established procedures for the synthesis of bulk imide-linked COFs with reasonable framework crystallinity: (i) the solvothermal approach introduced by Yan et al. in which the precursors are reacted in a mixture of the high boiling solvents mesitylene/NMP in varying ratios together with a catalytic base, namely, isoquinoline at temperatures between 200 and 250 °C for several days;^13,14,16−19^ (ii) the ionothermal approach, recently introduced by our group, where the linker molecules are mixed with ZnCl_2_, or with a ZnCl_2_ containing eutectic salt mixture.^15^ In the latter approach, while the reaction time could be reduced from days to only a few hours and the amount of environmentally harmful chemicals could be minimized, the use of temperatures as high as 250–300 °C could not be avoided.^15^

Unterlass and co-workers demonstrated that polycondensation under hydrothermal conditions can lead to crystalline 1D polyimides (PI).^20−22^ Although this method works well for 1D PIs, the adaptation of this hydrothermal synthetic strategy for 2D COFs is rather challenging. Following the work of Unterlass and co-workers, Kim and co-workers recently reported the synthesis of PIC-Ph COF under hydrothermal conditions.^23^ However, its applicability in synthesizing crystalline, high surface area COFs is restricted to the *p*-phenylenediamine linker, a water-soluble linker molecule. Using less water-soluble linker molecules such as 4,4′-diaminobiphenyl or 4,4′-diamino-*p*-terphenyl results in a limited long-range growth of the 2D structure and therefore in COFs with very low surface areas.^23^

We now report a general environmentally friendly approach for the synthesis of imide-linked COFs by alcohol-assisted hydrothermal polyimide condensation (aaHTP). We investigate the factors affecting the COF formation under aaHTP conditions thoroughly and optimize the synthetic protocol with respect to sustainability. Three COFs were synthesized with high long-range order and porosity of which one, the TAPE-PMDA-COF, crystallizes in a *kagome*-type lattice and could only be synthesized with sufficient crystallinity using the aaHTP protocol.

Another strategy to realize otherwise inaccessible COFs is to employ reported COFs as templates and modify them by postsynthetic modification or linker exchange. For instance, Yaghi et al.^24,25^ and others^7,26,27^ used the chemical conversion method to modify the linkage of COFs postsynthetically. Dichtel et al.^28^ and others,^17,29−31^ on the other hand, performed COF-to-COF or COP-to-COF transformations via monomer exchange reactions.

In this manuscript, we demonstrate that an imine-linked COF can be transformed into an imide-linked COF via linkage replacement using a sustainable hydrothermal polyimide condensation strategy, thus enabling unique access to imide-linked COFs inaccessible using solvothermal, ionothermal, and direct hydrothermal routes.

## Results and Discussion

**Scheme 1:**
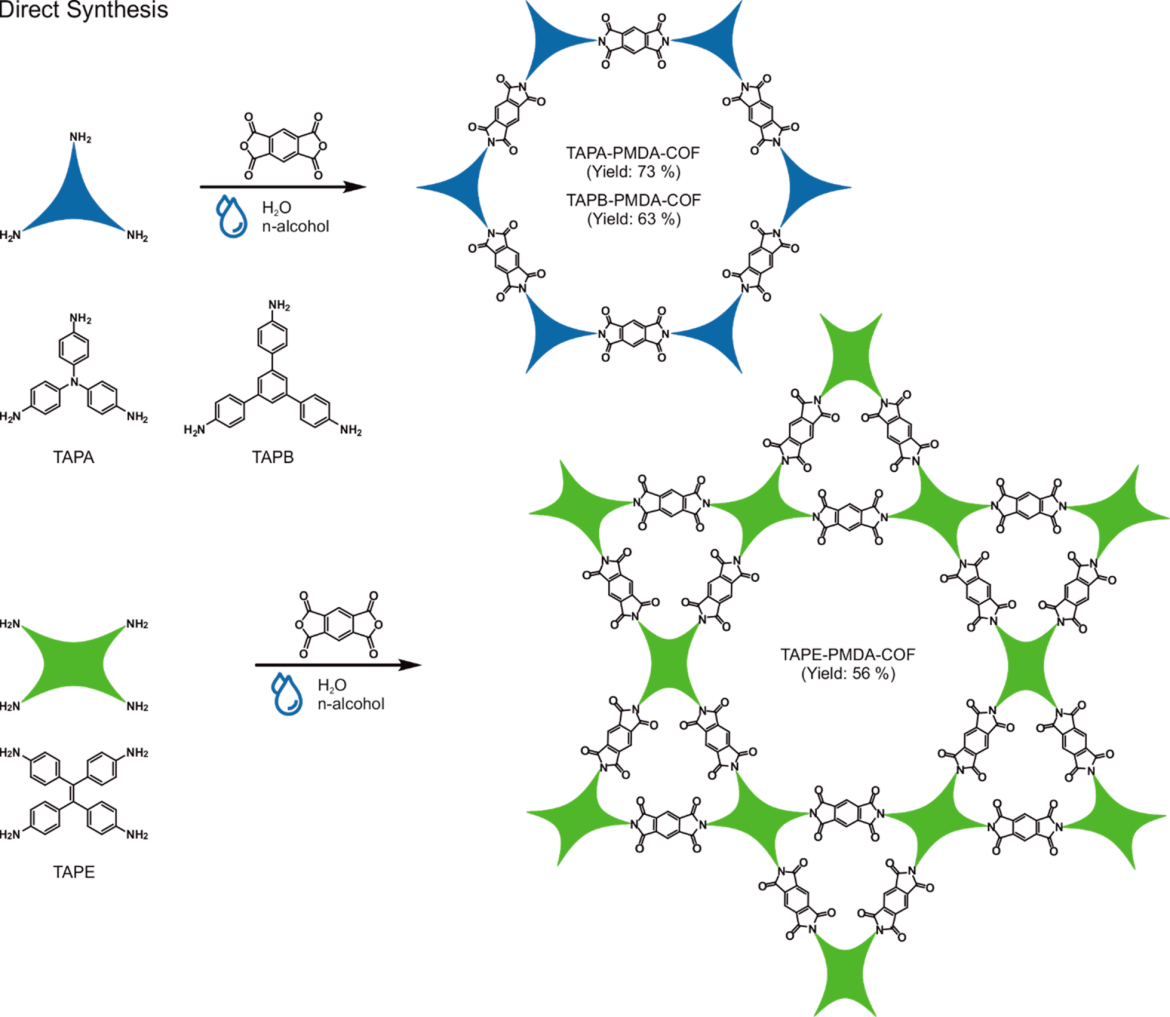
Direct Synthesis of Imide-Linked COFs from the Respective Amine Precursor Molecules TAPA, TAPB, and TAPE and the Anhydride Precursor Molecule PMDA *via* Direct aaHTP

We started our research with the literature-known TAPA-PMDA-COF and TAPB-PMDA-COF, which are synthesized from the precursor molecules tris(4-aminophenyl)amine (TAPA), 1,3,5-tris(4-aminophenyl)benzene (TAPB), and pyromellitic dianhydride (PMDA; Scheme 1).^13^

First attempts to synthesize these imide-linked COFs in water only were unsuccessful and yielded amorphous PIs (Figure S1). Interestingly, the imide polymerization itself seemed to occur in the presence of water as evident from the typical imide vibrational bands in the fourier transform infrared (FT-IR) spectra of the precipitate (Figure S1). We thus reasoned that crystallization may either be prevented by a lack of solubility of the amine precursor molecules and imide oligomers in water,^23^ hindering the growth of well oriented COF sheets, or the lack of polarity differences in the reaction mixture. Indeed, most COFs have been reported to form crystalline structures in two-solvent mixtures of varying polarity, e.g., mesitylene (ε = 2.4)^32^ and NMP (ε = 32)^33^ for imide-linked COFs.^34^ One possible reason could be the dielectric stabilization of oligomers or agglomerates in solution, keeping them accessible during the reaction. Further, the reversibility of the imide condensation reaction in water alone might not be sufficient enough for the defect healing processes needed to form well oriented 2D COF sheets in accordance with the principles of dynamic covalent chemistry.^1^

With this in mind, and with an aim to keep the reaction conditions as environmentally friendly as possible, we added n-alcohols of different chain lengths to the reaction mixture of the TAPA-PMDA-COF, together with catalytic amounts of pyridine (40 μL), and analyzed the received products. n-Alcohols were chosen because of the less toxic nature of this solvent class as compared to other organic solvents.^35^ Pyridine was added as a basic and nucleophilic catalyst and substitutes isoquinoline that is commonly used in solvothermal approaches.^13^ While no precipitation occurred in the reaction mixture with methanol and only an amorphous polymer could be isolated using ethanol, the reaction mixture with n-propanol yielded crystalline TAPA-PMDA-COF (Figure S2). (The complete characterization of TAPA-PMDA-COF, synthesized under optimized conditions, is presented later.)

**Figure 1:**
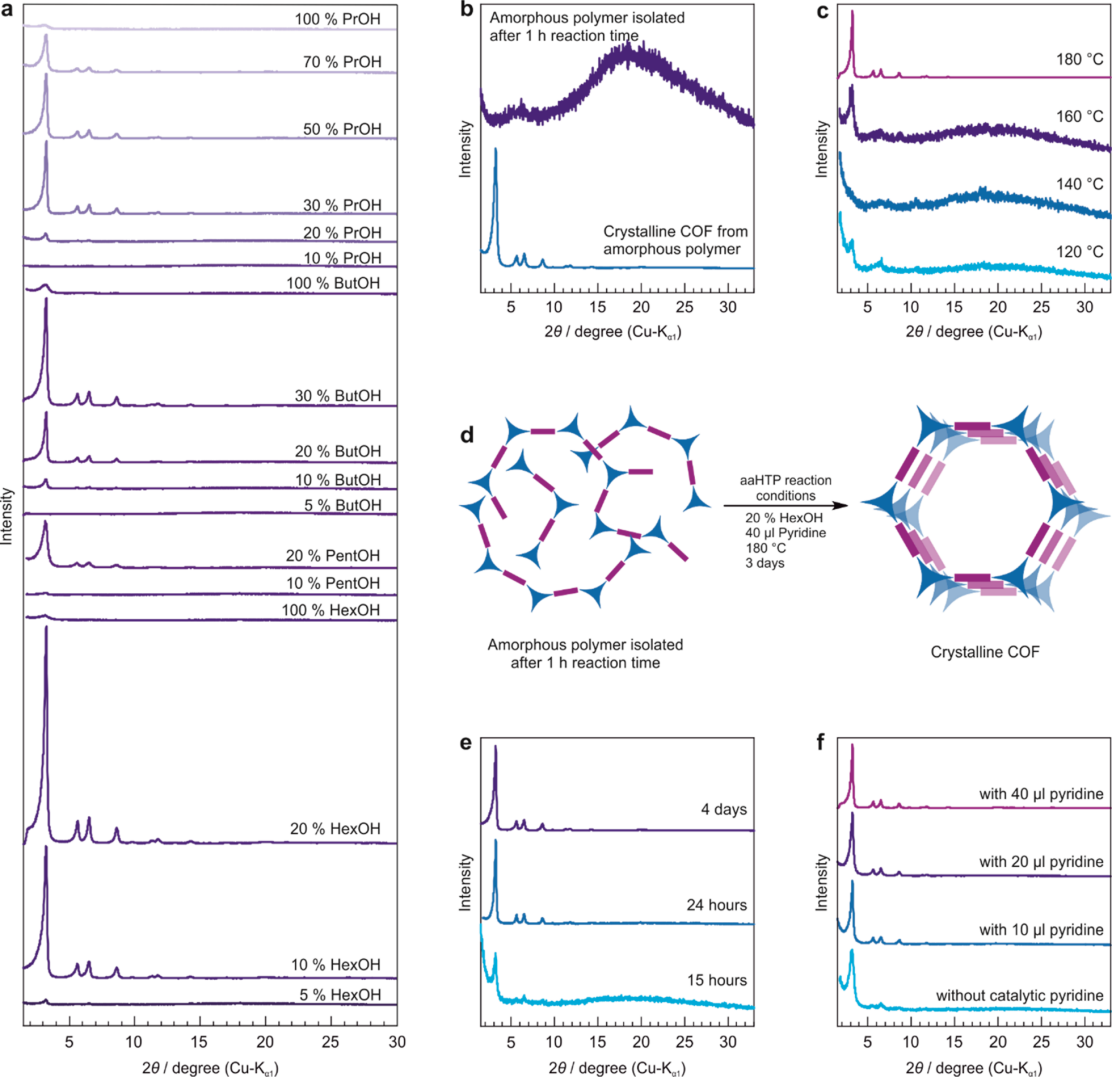
Optimization of the aaHTP protocol with TAPA-PMDA-COF as a model system. XRPD patterns of TAPA-PMDA-COF synthesized using differing conditions showing (a) the effect of the type and the amount of the n-alcohol on aaHTP, (b, d) the recrystallization of the amorphous PI into the crystalline COF under aaHTP conditions, (c) the effect of different reaction temperatures, (e) the effect of different reaction times, and (f) the COF synthesized using varying amounts of catalytic pyridine.

Motivated by the results and to get further insights into the factors affecting the aaHTP for imide-linked COFs, we tested a series of different n-alcohols in various concentrations and pyridine in the reaction mixture using TAPA-PMDA-COF as the model system. Additionally, to further reduce the energy expenditure of the aaHTP, we tested if a reduction of the reaction temperature and time is possible. The results of these investigations are depicted in Figure 1. Figure 1a shows the X-ray powder diffraction (XRPD) patterns of TAPA-PMDA-COF synthesized using different n-alcohols, ranging from n-propanol to n-hexanol, with varying concentrations. It can be seen that, while with all of the tested n-alcohols crystalline products form, the concentration of alcohol needed to produce crystalline products follows a trend: With increasing length of the alkane chain of the n-alcohol, and thereby increasing polarity difference in the solvent mixture (Figure S3), the amount of alcohol needed to get crystalline products is reduced. For example, while with n-propanol the alcohol content of the reaction mixture can only be reduced to 30% in order to obtain a crystalline product, with n-butanol a reduction to 20% is possible. Further, with n-hexanol, a reduction of the alcohol content to 10% could be achieved for the synthesis of TAPA-PMDA-COF. Ar sorption experiments of the most promising samples revealed that the BET surface area follows roughly the same trend as crystallinity (Figure S4): The higher the polarity difference of the solvent system, the higher the measured BET surface area. However, there are “sweet spots” for the water/n-alcohol ratios for each of the tested n-alcohols. While COFs synthesized in 20% n-hexanol or 20% n-butanol have calculated BET surface areas of 1062 m^2^/g and 244 m^2^/g, respectively, by varying the alcohol content by only 10% the surface areas increase to 1619 m^2^/g (10% n-hexanol) and 1327 m^2^/g (30% n-butanol).

It should also be noted that the synthesis of TAPA-PMDA-COF in 100% alcohol did not lead to crystalline products in any case, indicating that the interplay between the respective alcohol and water is crucial for the hydrothermal imide-COF formation.

To further elucidate the role of the alcohol in the aaHTP, two COF reactions were performed in which one reaction was carried out in water and pyridine and the other one in water, 30% butanol, and pyridine. Both experiments were done simultaneously, and multiple images were taken during the process of heating to 200 °C (Figure S5). After 1 h at 200 °C, the reaction mixtures were allowed to cool down to approximately 100 °C in order to reduce the inside pressure, and the supernatant was filtered off hot and analyzed via FT-IR and ^1^H nuclear magnetic resonance (NMR) spectroscopy. Additionally, the precipitate was analyzed via FT-IR spectroscopy and XRPD. During this experiment, two observations were made: (1) In both cases, the precipitation of the amorphous TAPA–PMDA–PI occurred rapidly and already during the heating process (Figure S6). This indicates recrystallization of the amorphous PI after precipitation to form the final COF. This could indeed be confirmed by heating the isolated amorphous polymer a second time under aaHTP conditions for 3 days without adding additional precursor molecules, which resulted in the crystalline TAPA-PMDA-COF (Figure 1b,d). (2) While in the supernatant of the alcohol containing reaction mixture imide and amic acid species could be detected, the supernatant of the water reaction mixture contained predominantly deprotonated pyromellitic acid and protonated amine linker molecules (Figures S7–S10). This indicates the formation of a monomer-salt intermediate in the COF formation reaction prior to polymerization, by protonation of the amine linker by the hydrolyzed PMDA as described previously.^20,23^ We observed that this monomer-salt intermediate (Figure S11) dissolves in water and also in the water/n-alcohol mixtures during the heating process, and amorphous PI begins to precipitate thereafter. Thus, while water could serve as a good solvent for PMDA and the intermediate salt, enabling the primary diffuse polymerization, and enhances the reversibility of the equilibrium reaction (Le Chatelier’s principle), the alcohol could contribute to enhancing the solubility of the amine precursor, enabling exchange of the amine linker molecule from the precipitated amorphous network during the defect healing processes. Indeed, solubility experiments with TAPA in pure water and in a series of n-alcohols (Figure S12) revealed that it is barely soluble in pure water, even at 180 °C, whereas it dissolves completely in all alcohols tested.

In addition, the less polar imide dimers and oligomers, detected in the supernatant of the alcohol-containing reaction, are possibly kept longer in solution due to a surfactant-like behavior of the longer chain n-alcohols which can interact with the less polar benzylic groups of the oligomers in a manner similar to that observed in surfactant-based MOF formation.^36^ This could explain the requirement of progressively smaller alcohol concentrations as one moves from n-propanol to n-hexanol and provides a great advantage over the pure hydrothermal synthesis approach, circumventing the need for water-soluble linker molecules and oligomers to achieve high quality COFs.^23^

Figure 1c shows the XRPD pattern of TAPA-PMDA-COF synthesized at different reaction temperatures ranging from 120 to 180 °C in 10% n-hexanol/pyridine for 4 days. It can be seen that a reduction of the reaction temperature to <180 °C results in a significant loss of crystallinity. This shows that a certain reaction temperature and therefore also a certain autogenous pressure (approximately 12 bar) of the reaction mixture is required to ensure sufficient reversibility of the imide condensation reaction. It is hence not possible to reduce the energy expenditure of the aaHTP by lowering the reaction temperature. Nevertheless, as shown in Figure 1e, we were able to reduce the reaction time for the synthesis of TAPA-PMDA-COF to 24 h. A further reduction of the reaction time led to a significant loss of crystallinity. However, the calculated BET surface area of 603 m^2^/g after 24 h indicates that further time is required for the COF crystallization to be completed (Figure S13).

Since the most toxic substance used in the aaHTP is pyridine, we tried to reduce its usage and tested additionally if the reaction yields crystalline products when using lower amounts of this catalyst or in the absence of pyridine. As depicted in Figure 1f, the samples synthesized with reduced amounts of pyridine (20% n-hexanol, 180 °C, 4 days) are crystalline. However, with decreasing pyridine content, we observed a progressive broadening of the reflections and a reduction of the BET surface areas (Figure S14). The sample synthesized without pyridine shows broad reflections as well, and the poor signal intensities point toward the formation of only a few small crystalline domains due to the reduced reversibility. Argon sorption measurements of this sample revealed a BET surface area of only 320 m^2^/g (Figure S14), corroborating this argument.

Nevertheless, with TAPA-PMDA-COF, it could be demonstrated that the aaHTP at 180 °C, in a reaction mixture consisting of 90% water, leads to crystalline imide-linked COFs, thus making this method substantially more sustainable than the commonly used solvothermal or ionothermal approach for imide-linked COFs.^13,15^ Additionally, the water treatment after the reaction is simplified with this method because the organic phase consisting of either n-butanol or n-hexanol and pyridine can easily be separated from the aqueous phase after the COF synthesis due to phase separation at room temperature. The separated organic phase can be recycled and used for further synthesis of the same COF (Figure S15).

With the knowledge gained from optimizing the synthesis of TAPA-PMDA-COF, we were able to adapt the aaHTP to synthesize two additional imide-linked COFs directly from their respective linker molecules. TAPB-PMDA-COF and TAPE-PMDA-COF have been synthesized from the precursor molecules TAPB, 1,1,2,2-tetrakis(4-aminophenyl)ethylene (TAPE), and PMDA (Scheme 1). While TAPB-PMDA-COF has previously been known,^13^ TAPE-PMDA-COF has not been reported so far. Attempts to synthesize TAPE-PMDA-COF using known literature methods failed (Figure S16), showing the importance of the development of new synthetic protocols and hence of the aaHTP.^13,15^ The optimized reaction conditions have been found to be 90% H_2_O/10% n-hexanol/pyridine for TAPA-PMDA-COF, 80% H_2_O/20% n-hexanol/pyridine for TAPB-PMDA-COF, and 67% H_2_O/33% n-butanol/pyridine for TAPE-PMDA-COF.

**Figure 2:**
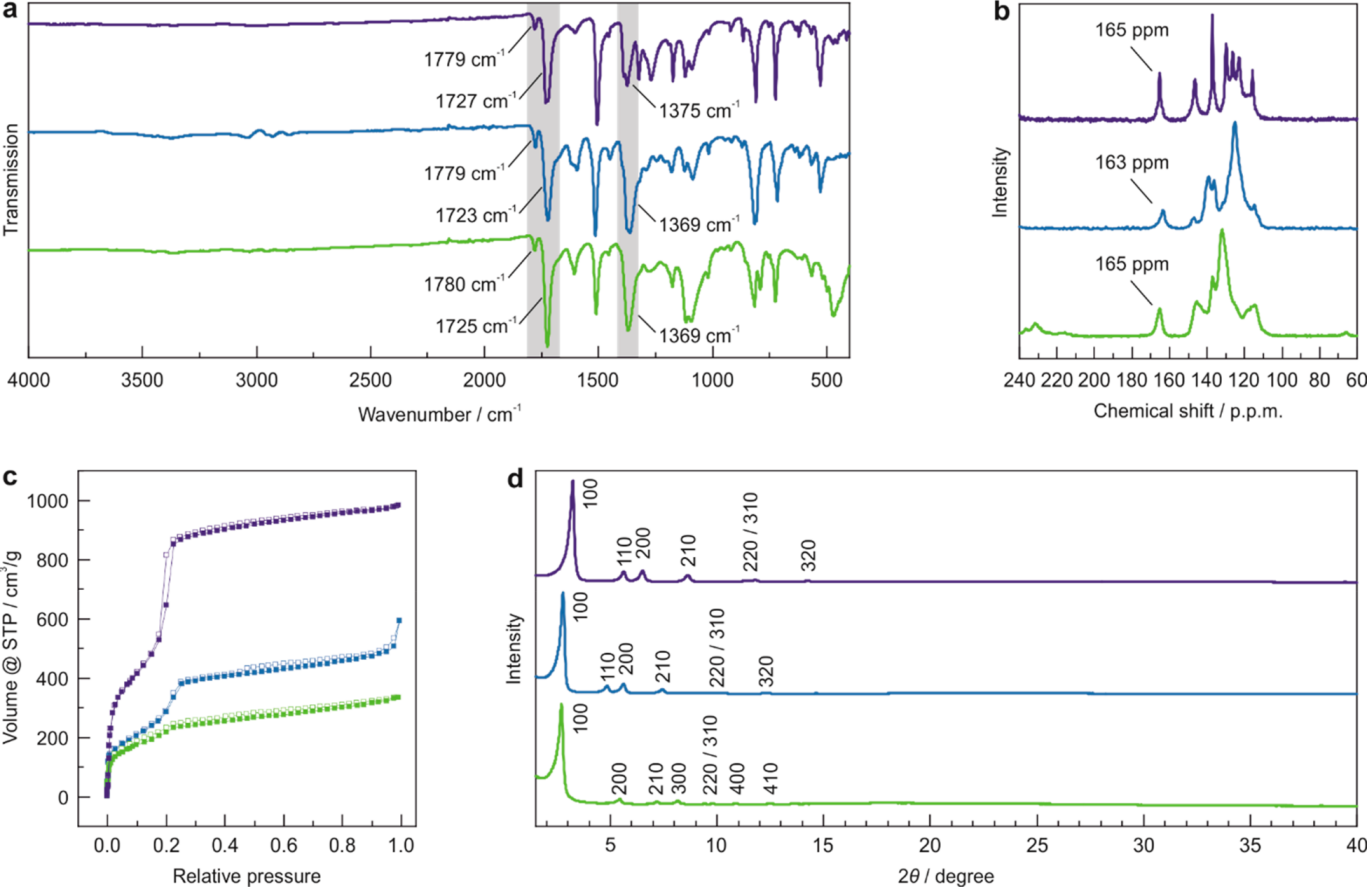
Characterization of TAPA-PMDA-COF (violet), TAPB-PMDA-COF (blue), and TAPE-PMDA-COF (green). (a) Normalized FT-IR spectra showing the characteristic C=O and C–N–C vibrations of the imide-ring. (b) ^13^C ssNMR spectra showing the chemical shifts of the imide ring carbon at 165 ppm. (c) Ar adsorption (filled symbols)/desorption (hollow symbols) isotherms. (d) Experimental XRPD patterns normalized to the highest intensity 100 Bragg peak.

FT-IR, ^13^C CP-MAS solid-state NMR (ssNMR), XRPD, and argon sorption measurements of the resulting solids corroborated the formation of the COFs (Figure 2). FT-IR spectra of the COFs show the presence of imide vibrational bands with the appearance of the characteristic antisymmetric and symmetric C=O stretching vibrations at around 1779 and 1725 cm^–1^ and the C–N–C stretching vibration at around 1369 cm^–1^ (Figure 2a). Moreover, the absence of the characteristic vibrational bands of the anhydride (1700 cm^–1^) and amine (3367 cm^–1^) functional groups in the FT-IR spectra point to the completeness of the reaction (Figure S17). ^13^C ssNMR further confirmed the formation of the imide linkage with the characteristic carbonyl carbon of the imide ring appearing at around 165 ppm (Figure 2b). All three COFs are crystalline, as evident from their respective XRPD patterns (Figure 2d) and TEM images (Figure S18). Rietveld^37^ refinement of the experimental powder diffraction patterns (Figures S19, S20) yielded the unit cell parameters *a* = 31.06 Å and *c* = 3.91 Å for TAPA-PMDA COF (*P*622 symmetry) and *a* = 36.04 Å and *c* = 3.75 Å for TAPB-PMDA-COF (*P*31*m* symmetry). TAPE-PMDA-COF was simulated with *P*622 symmetry and *kagome*-type structure. Rietveld refinement yielded the unit cell parameters *a* = 37.46 Å and *c* = 5.00 Å (Figures S19, S20). The porosity of the hydrothermally synthesized COFs was confirmed by argon sorption measurements at 87.15 K from which BET surface areas of 1619 m^2^/g (TAPA-PMDA-COF), 939 m^2^/g (TAPB-PMDA-COF), and 689 m^2^/g (TAPE-PMDA-COF) were calculated (Figures 2c, S21). The calculated pore size distribution (PSD) of TAPE-PMDA-COF shows a dual pore system with pore sizes of 1.67 and 2.66 nm, further confirming the *kagome*-type structure with a dual pore system. Note that the BET surface area of TAPA-PMDA-COF is the highest reported so far for this system and that the value for TAPB-PMDA-COF compares to previously reported values for this COF synthesized either under standard solvothermal conditions using high boiling organic solvents or under ionothermal conditions at high temperatures.^13,15,38−40^

As an alternative strategy to the direct aaHTP for the synthesis of imide-linked COFs, we explored if the aaHTP protocol can be adapted in a possible linker exchange strategy for the synthesis of otherwise inaccessible imide-linked COFs from imine-linked COFs. As shown for other COF systems, the exchange of linker molecules in a COF relies on the requirement that the replaced linkage is more reversible and less stable under the chosen reaction conditions, while the substituting linkage has to be less reversible and more stable.^17,28−30,41^ In addition, the preorientation through—or template function of—the starting COF, and a reduced reaction speed when compared to the direct synthesis from the respective precursor molecules, assist the formation of crystalline products after exchange.

As the model precursor COF system for the imine-linked to imide-linked COF transformation using the aaHTP method, we chose imine-linked Py1P-COF for its transformation to Py-imide COF, previously unreported and inaccessible using all known synthetic methods including direct aaHTP (Figure S22). The lack of crystallinity when using direct aaHTP could be explained due to a reduced solubility of the large amine linker in the n-alcohol/H_2_O mixture compared to the smaller linker molecules used successfully in direct aaHTP (Figures S23, S24). Using the linkage exchange strategy, we take advantage of the preorientation of the imine COF and additionally have a potential modulating effect through the released aldehyde linker during the reaction. The model system was chosen because the exchanging linker molecules terephthaldehyde and PMDA are nearly equally sized (Figure S25).

**Scheme 2:**
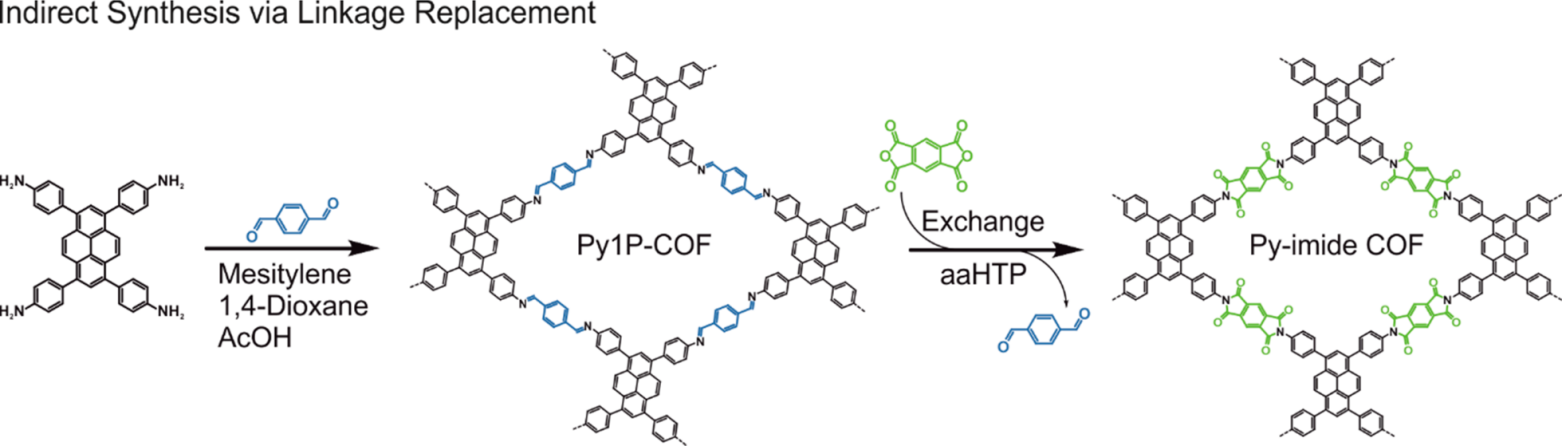
Synthesis of the Imine-Linked Precursor Py1P-COF and Its Transformation into the Imide-Linked Py-Imide COF via Linkage Replacement Using aaHTP Conditions

**Figure 3:**
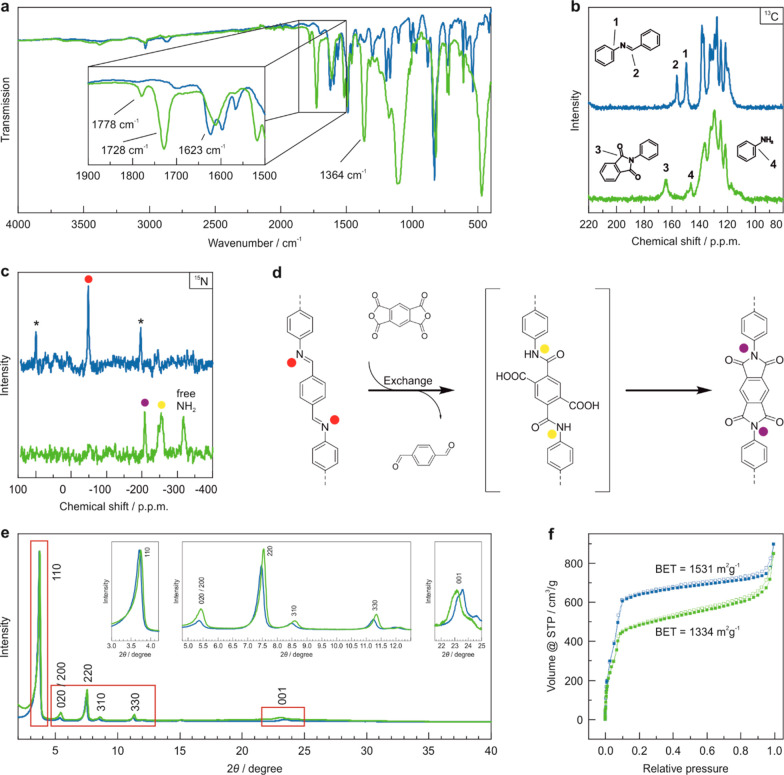
Characterization of Py1P-COF (blue) and Py-Imide COF (green). (a) FT-IR spectra showing the appearance of the characteristic imide vibrational bands and the disappearance of the imine C=N vibration. (b) ^13^C ssNMR spectra demonstrating the conversion of the imine linkage to the imide linkage. (c) ^15^N ssNMR spectra showing the successful conversion into the Py-imide COF and revealing intermediate amic acid residuals. (d) Scheme of the conversion of the imine linkage to the imide linkage together with the assignments of the ^15^N ssNMR spectra. (e) Overlay of the COFs’ experimental XRPD pattern normalized to the highest intensity 110 reflection together with enlargements of the COFs’ XRPD patterns to visualize the differences in relative intensity and reflection position upon transformation. (f) Ar adsorption (filled symbols)/desorption (hollow symbols) confirming the retention of porosity.

Py1P-COF, synthesized following a reported procedure from the precursor molecules 4,4′,4″,4′′′-(pyrene-1,3,6,8-tetrayl)tetraaniline (PyTTA) and terephthalaldehyde in a mixture of mesitylene/1,4-dioxane/6 M AcOH (Scheme 2),^42^ was reacted with PMDA in a mixture of 67% H_2_O/33% n-butanol/0.04 mL of pyridine for 4 days. FT-IR spectroscopy of the resulting brown-orange powder reveals vibrational bands at 1778, 1728, and 1364 cm^–1^, which can be assigned to the characteristic antisymmetric and symmetric C=O stretching vibrations and the C–N–C stretching vibration of the imide ring of Py-imide COF (Figure 3a). The characteristic N=CH vibrational band at 1623 cm^–1^ of the imine linked Py1P-COF vanishes, indicating the successful transformation into the Py-imide COF. ^13^C and ^15^N CP-MAS ssNMR spectroscopy further confirm the conversion of Py1P-COF to Py-imide COF as evident by the disappearance of the signals for carbon **1** and **2** at 156.3 and 149.6 ppm (Figure 3b) and the appearance of the signal for carbon **3** at 164.5 ppm, as well as the disappearance of the imine nitrogen at −49 ppm (red dot) and the appearance of the imide nitrogen at −208 ppm (Figure 3c,d). Two additional signals at −254 ppm and −318 ppm appear in the ^15^N spectrum of the Py-imide COF. These signals can be assigned to unreacted or dangling amine functional groups and to the amic acid intermediate described previously for the imide-COF condensation reaction (Figure 3c,d).^43^ Note that the ssNMR spectra are measured using N–H cross-polarization methods and are therefore not quantitative.

The signals of nitrogen atoms connected to hydrogen atoms appear more intense than those without hydrogen atoms. Since there is no indication for greater amounts of amic acid or imine residuals in the ^13^C ssNMR spectrum (peak missing at 173 ppm), we assume an almost complete condensation. However, a peak at 146 ppm appears in the ^13^C spectrum, which corresponds to carbon **4**, revealing an increased amount of free amine groups. Further, argon sorption measurements indicate that the porosity of the structure is largely retained after transformation and reveals only a small change of the BET surface area from 1531 m^2^/g to 1334 m^2^/g (Figures 3f, S26). Pore size distribution (PSD) analysis of the Py-imide COF using the QSDFT method reveals pores of 2 nm for Py-imide COF, in accordance with the theoretical pore size of 2 nm for the refined crystal lattice (*vide infra*).

The experimental XRPD patterns (Figure 3e) and TEM images (Figure S27) of the precursor Py1P-COF and the product Py-imide COF show high crystallinity and reveal that the framework remains intact upon transformation. The diffraction patterns of the two COFs look very similar, which is expected since the geometry and the size of the unit cell before and after conversion should not differ significantly due to the nearly equally sized linker molecules terephthaldehyde and PMDA. Py1P-COF shows reflections at 3.71, 5.36, 7.47, 8.47, 11.24, and 23.44° 2θ, and Py-imide COF shows reflections at 3.75, 5.43, 7.54, 8.59, 11.34, and 23.1° 2θ. It can be observed that all reflections shift slightly toward higher scattering angles, except the 001 stacking reflection at around 23° 2θ, which shifts toward lower angles. This indicates a slight decrease of the unit cell parameters in the *a*–*b* plane and a simultaneous increase of the stacking distance in the [001] direction. Further, it can be observed that the intensity of the reflections between 5 and 12° 2θ increases upon transformation, which is most likely an effect of an increasing layer offset after linker exchange as can be seen by the differing β values.^44^ The experimental XRPD patterns are in good agreement with simulated reflection patterns using *C*2/*m* symmetry for both COFs, suggesting a topotactic transformation (Figures S28, S29). Rietveld refinement of the XRPD patterns yield unit cell parameters of *a* = 38.06 Å, *b* = 32.47 Å, *c* = 3.82 Å, α = γ = 90°, and β = 63.3° for Py1P-COF and *a* = 33.87 Å, *b* = 32.16 Å, *c* = 3.9 Å, α = γ = 90°, and β = 86.7° for Py-imide COF.

Stability tests were then performed (Figure S30) to ensure a successful exchange of the PMDA linker molecule into the COFs framework. While both COFs show high stability under strongly acidic conditions (conc. HCl, 60 °C, 24 h), there is a distinct difference between the two COFs under reductive conditions (50% HCOOH, 60 °C, 24 h). Py1P-COF partially dissolves, and the remaining solid turns amorphous. On the other hand, Py-imide COF shows high stability under these conditions with full retention of its crystalline structure. The stability of Py-imide COF over Py1P-COF under reductive conditions further substantiates a successful transformation of the latter to the former under aaHTP reaction conditions.

## Conclusion

We report an environmentally friendly alcohol-assisted hydrothermal polymerization approach (aaHTP) for the synthesis of imide-linked COFs. Using this method, we were able to synthesize TAPA-PMDA-, TAPB-PMDA-, and the previously unreported TAPE-PMDA-COF directly from their respective linker molecules. TAPE-PMDA-COF, which crystallizes in a *kagome*-type structure, could only be synthesized using the aaHTP protocol, showing the importance of such complementary procedures for the synthesis of COFs. The aaHTP is special in that a wide range of imide-linked COFs can now be synthesized in reaction mixtures consisting of up to 90% water, irrespective of the water solubility of their linker molecules, making this an eco-friendly and general alternative method to all previously reported synthetic procedures. Additionally, using the example of the newly synthesized imide-linked Py-imide COF, we demonstrate the applicability of aaHTP in COF-to-COF transformations via linkage replacement from imine-linked COFs in an environmentally friendly fashion enabling access to imide-linked COFs that were inaccessible before. This work thus provides a simple and low-cost synthesis strategy for imide-linked COFs and presents a substantial contribution to the field of green chemistry in general and to the field of sustainable COF synthesis in particular.

## Experimental Section

### Materials

4,4′,4″,4′′′-(pyrene-1,3,6,8-tetrayl)tetraaniline was synthesized according to literature procedures.^27,42^ All other chemicals were obtained from commercial sources and were used as received.

### Methods

All hydrothermal experiments were carried out in “Schlenk bombs”. Schlenk bombs are a subclass of Schlenk flasks of structurally sound shapes and heavy walls that have only one opening which is accessed by opening a Teflon plug valve. Due to the Teflon plug valve, Schlenk bombs can be sealed more completely than standard Schlenk flasks. This design allows reactions at elevated temperatures and pressures.

### Synthesis of TAPA-PMDA-COF

Tris(4-aminophenyl)amine (TAPA, 0.067 mmol, 19.4 mg) and pyromellitic dianhydride (PMDA, 0.1 mmol, 21.8 mg) were placed in a Schlenk bomb and suspended in a mixture of 1.8 mL of H_2_O/0.2 mL of n-hexanol/0.04 mL of pyridine (or 1 mL of H_2_O/0.5 mL of n-butanol/0.04 mL of pyridine). The reaction mixture was sonicated for 10 min and subsequently degassed by four freeze–pump–thaw cycles. The Schlenk bombs were placed in an aluminum heating block and heated to 180 °C (∼12°bar) for 4 days. The resulting brown precipitate was isolated via filtration and washed with methanol and THF followed by Soxhlet extraction in methanol. Supercritical CO_2_ drying yielded TAPA-PMDA-COF in 73% yield.

### Synthesis of TAPB-PMDA-COF

Tris(4-aminophenyl)benzene (TAPB, 0.067 mmol, 23.4 mg) and pyromellitic dianhydride (PMDA, 0.1 mmol, 21.8 mg) were placed in a Schlenk bomb and suspended in a mixture of 1.2 mL of H_2_O/0.3 mL of n-hexanol/0.04 mL of pyridine. The reaction mixture was sonicated for 10 min and subsequently degassed by four freeze–pump–thaw cycles. The Schlenk bombs were placed in an aluminum heating block and heated to 200 °C (∼16 bar) for 6 days. The resulting brown precipitate was isolated via filtration and washed with methanol and THF followed by Soxhlet extraction in methanol. Supercritical CO_2_ drying yielded TAPB-PMDA-COF in 63% yield.

### Synthesis of TAPE-PMDA-COF

1,1,2,2-tetrakis(4aminophenyl)ethylene (TAPE, 0.05 mmol, 19.6 mg) and pyromellitic dianhydride (PMDA, 0.1 mmol, 21.8 mg) were placed in a Schlenk bomb and suspended in a mixture of 1 mL of H_2_O/0.5 mL of n-butanol/0.04 mL of pyridine. The reaction mixture was sonicated for 10 min and degassed by four freeze–pump–thaw cycles. The Schlenk bombs were placed in an aluminum heating block and were heated to 190 °C (∼13 bar) for 4 days. The resulting brown precipitate was isolated via filtration and washed with methanol and THF followed by Soxhlet extraction in methanol. Supercritical CO_2_ drying yielded TAPB-PMDA-COF in 56% yield.

### Synthesis of Py1P-COF

Py1P-COF was synthesized according to a literature procedure:^42^ 4,4′,4″,4′′′-(pyrene-1,3,6,8-tetrayl)tetraaniline (0.06 mmol, 34 mg) and terephthal aldehyde (0.114 mmol, 15.6 mg) were placed in a microwave vial and suspended in a mixture of 2 mL of mesitylene/1 mL of 1,4-dioxane and 0.3 mL of 6 M AcOH. The reaction vessel was closed and heated to 120 °C for 4 days. The bright orange Py1P-COF was isolated via filtration and washed with THF and DCM, followed by Soxhlet extraction with methanol. Supercritical CO_2_ drying yielded Py1P-COF in 79.7% yield.

### Transformation of Py1P-COF into Py-imide-COF

Py1P-COF (0.04 mmol, 30 mg) and pyromellitic dianhydride (0.16 mmol, 34.9 mg) were placed in a Schlenk bomb and suspended in a mixture of 1.0 mL of H_2_O/0.5 mL of n-butanol/0.04 mL of pyridine. The reaction mixture was sonicated for 10 min and subsequently degassed by four freeze–pump–thaw cycles. The Schlenk bombs were placed in an aluminum heating block and heated to 200 °C (∼16 bar) for 4 days. The resulting brown orange precipitate was isolated via filtration and washed with methanol, THF, and ethanol. Still wet, the solid was suspended in a mixture of ethanol and formic acid (1:1) at 60 °C for 18 h to remove potential unreacted Py1P-COF. The solid was filtered and washed again with THF and methanol, followed by Soxhlet extraction with methanol. Supercritical CO_2_ drying yielded Py-Imide COF in 92% yield.

## Supporting Information Available

Additional analytical method descriptions and data including XRPD pattern, FT-IR spectra, sorption isotherms, TEM images, and optimized structures (PDF)
