# Supplementary material for: Direct and Linker-Exchange Alcohol-Assisted Hydrothermal Synthesis of Imide-Linked Covalent Organic Frameworks
Source: Chem Mater. 2022 Feb 17;34(5):2249–58. doi: 10.1021/acs.chemmater.1c04051 (PMC8908547; doi:10.1021/acs.chemmater.1c04051)
Supplement: Supplementary file 1 — cm1c04051_si_001.pdf [file cm1c04051_si_001.pdf]

Supporting Information

Direct and Linker-Exchange Alcohol-Assisted Hydrothermal Synthesis of Imide-Linked Covalent Organic Frameworks

Johannes Maschita,[1,2] Tanmay Banerjee,[3] and Bettina V. Lotsch* [1,2,4]

[1] Max Planck Institute for Solid State Research, Heisenbergstraße 1, 70569 Stuttgart, Germany

[2] Department of Chemistry, University of Munich (LMU), Butenandtstraße 5-13, 81377 München, Germany

[3] Department of Chemistry, BITS Pilani, Pilani Campus, Rajasthan – 333031, India

[4] E-conversion and Center for Nanoscience, Schellingstraße 4, 80799 München, Germany

Abstract: Covalent organic frameworks (COFs) are an extensively studied class of porous materials, which distinguish themselves from other porous polymers in their crystallinity and high degree of modularity, enabling a wide range of applications. However, the established synthetic protocols for the synthesis of stable and crystalline COFs, such as imide-linked COFs, often requires the use of high boiling solvents and toxic catalysts making their synthesis expensive and environmentally harmful. Herein, we report a new environmentally friendly strategy – an alcohol-assisted hydrothermal polymerization approach (aaHTP) for the synthesis of a wide range of crystalline and porous imide-linked COFs. This method allows us to gain access to new COFs and to avoid toxic solvents by up to 90 % through substituting commonly used organic solvent mixtures with water and small amounts of n-alcohols without being restricted to water-soluble linker molecules. Additionally, we use the aaHTP to demonstrate an eco-friendly COF-to-COF transformation of an imine-linked COF into a novel imide-linked COF via linkage replacement, inaccessible using published reaction conditions.

Table of Contents

Experimental Procedures .... - 2 -

FT-IR .... - 2 -

TEM .... - 2 -

Sorption .... - 2 -

XRPD .... - 2 -

NMR .... - 2 -

Supplementary Data .... - 3 -

References .... - 19 -

Author Contributions .... - 19 -

Experimental Procedures

FT-IR

Infrared spectra were recorded on a PerkinElmer UATR Two in attenuated total reflection (ATR) geometry equipped with a diamond crystal.

TEM

TEM was performed with a Philips CM30 ST (300kV, LaB6 cathode). The samples were prepared dry onto a copper lacey carbon grid (Plano). Images were recorded with a TVIPS TemCam-F216 CMOS camera. The program EM-Menu 4.0 Extended was used for analysis.

Sorption

Sorption measurements were performed on a Quantachrome Instruments Autosorb iQ MP with Argon at 87 K. The pore size distributions were determined from argon adsorption isotherms using the quenched solid-state density functional theory (QSDFT) for cylindrical pores in carbon model for argon at 87 K.

XRPD

X-ray powder diffraction (XRPD) measurements were performed on a Stoe Stadi-P diffractometer in Debye-Scherrer geometry with Cu-$K_{\alpha 1}$ radiation equipped with a Ge(111) primary monochromator. The glass capillaries (1 mm in diameter) were spun during data collection for an improved particle statistics. Rietveld[1] refinements of the different COF structures were performed using TOPAS V6. Model structures created by Material Studio were used for the Rietveld refinements with fixed atomic coordinates. The peak profile of the XRPD patterns was described by applying the fundamental parameter[2] approach as implemented in TOPAS. The background was modeled by Chebychev polynomials. The microstructure of the different COFs was modeled using microstrain (Lorentzian and Gaussian components).

NMR

Solid state nuclear magnetic resonance spectra (ssNMR) were recorded on a Bruker Avance III 400 MHz spectrometer (magnetic field 9.4 T). For ssNMR spectroscopy, the samples were packed in ZrO_2 rotors, and spun in a Bruker WVT BL4 double resonance MAS probe. The spinning rate was 12-14 kHz in ^{13}C measurements, and 6 kHz in ^{15}N experiments. A standard cross-polarization sequence with a ramped contact pulse was used for both nuclei. The duration of contact pulse was

2 ms for ^{13}C and 4 ms for ^{15}N. A total of 4096-8192 scans were routinely accumulated in ^{13}C experiments, and 80000 scans in the experiments with ^{15}N. All the measurements were performed under conditions of high-power broadband proton decoupling (SPINAL 64) with the spectral conditions being optimized for the shortest relaxation delay by measuring ^{1}H T_1 relaxation time. Chemical shifts were referenced relative to tetramethylsilane in ^{13}C (δ_{iso} = 0.0 ppm) and relative to nitromethane in ^{15}N (δ_{iso} = 0.0 ppm), with solid glycine as the secondary reference (δ_{iso} [^{15}N] = -347.54 ppm).

Supplementary Data

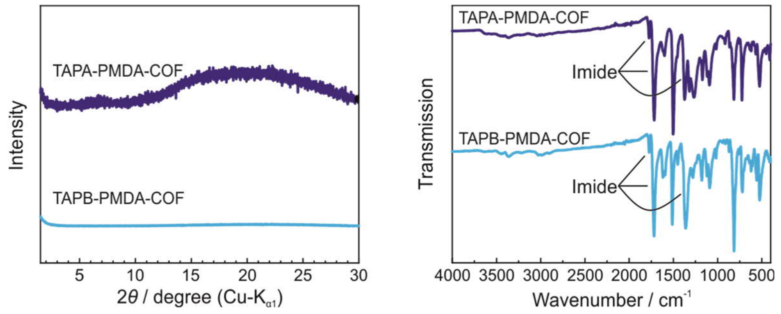

Figure S1. XRPD pattern (left) and FT-IR spectra (right) of the amorphous PI polymers TAPA-PMDA (violet) and TAPB-PMDA (blue) received from the synthesis in pure water at 200 °C.

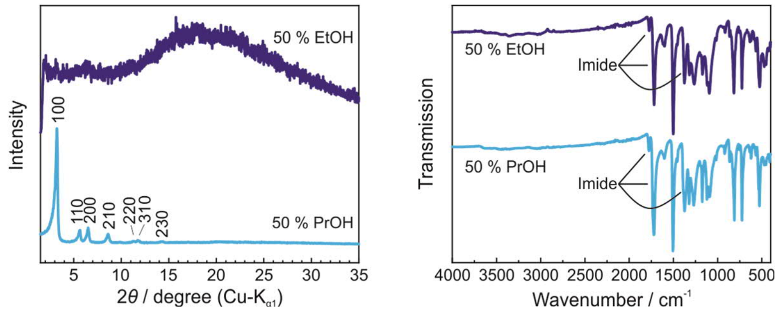

Figure S2. XRPD pattern (left) and FT-IR spectra (right) of TAPA-PMDA-COF samples synthesized in a mixture of 1 ml H_2O/1 ml ethanol/0.04 ml pyridine (violet) and 1 ml H_2O/1 ml n-propanol/0.04 ml pyridine (blue).

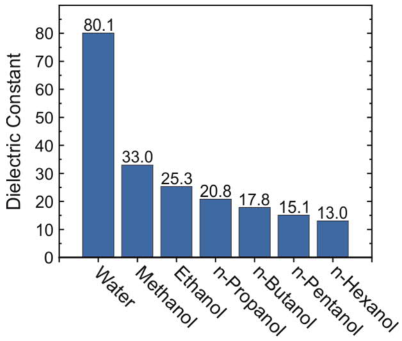

Figure S3. Dielectric constants of water compared to a row of n-alcohols from methanol to n-hexanol.[3]

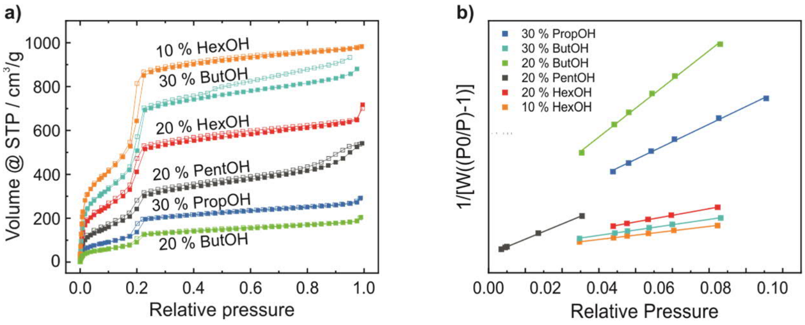

c)

Solvent	BET Surface Area
10 % HexOH	1619 m^2/g
20 % HexOH	1062 m^2/g
20 % PentOH	726 m^2/g
20 % ButOH	244 m^2/g
30 % ButOH	1327 m^2/g
30 % PropOH	397 m^2/g

Figure S4. a) Ar sorption and desorption isotherms of TAPA-PMDA-COF synthesized in H2O/n-alcohol/pyridine mixtures with varying alcohols and alcohol concentrations together with b) the corresponding BET plots and c) the calculated BET surface areas.

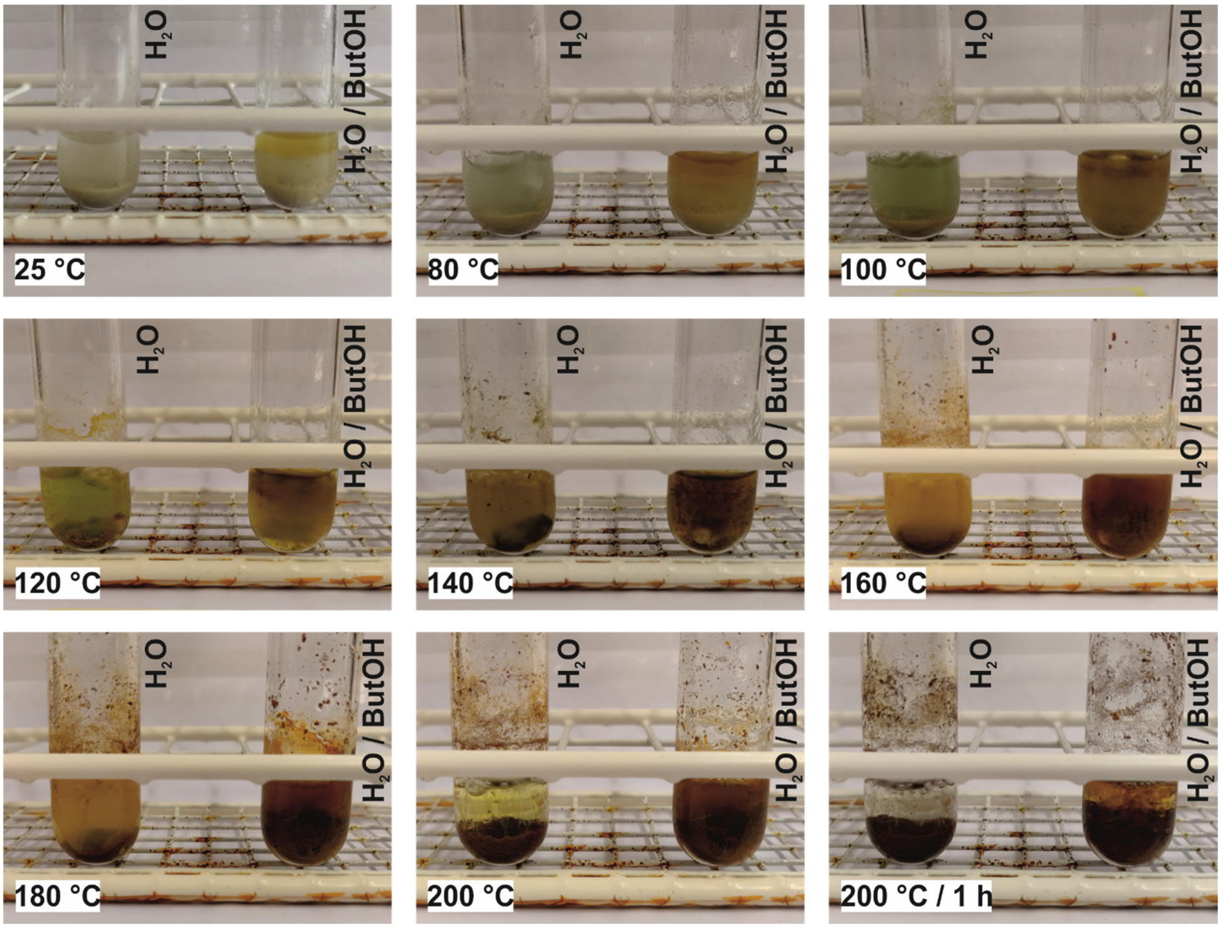

Figure S5. Pictures taken during the heating process of the TAPA-PMDA-COF reaction in 1.5 ml H_2O/0.04 ml pyridine (left tube) and 1.0 ml H2O/0.5 ml n-butanol/0.04 ml pyridine (right tube).

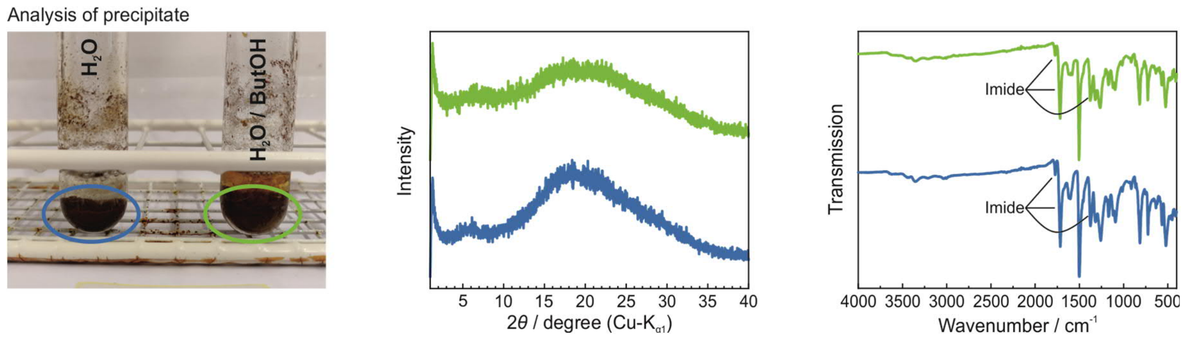

Figure S6. XRPD patterns (middle) and FT-IR spectra (right) of the precipitate of the TAPA-PMDA-COF reactions obtained from the reaction observation experiment in Figure S5 after 1 h at 200 °C (left). The analysis revealed that imide formation occurs rapidly already during the heating process in both cases, but in an amorphous fashion.

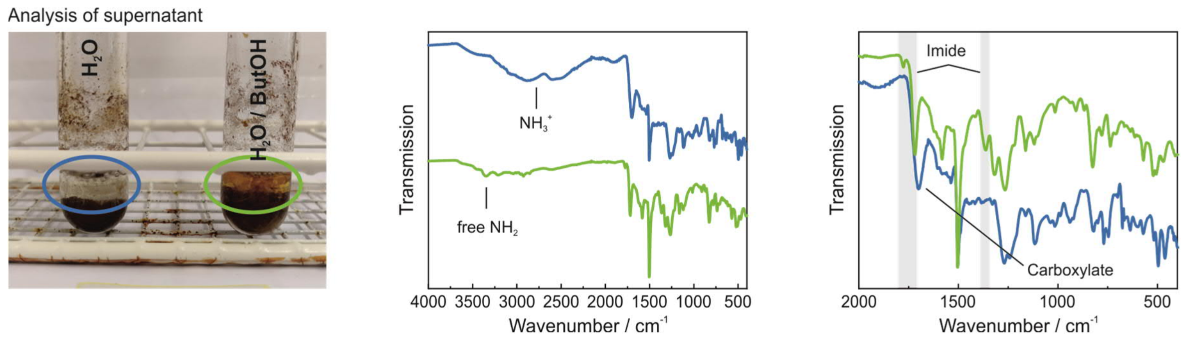

Figure S7. FT-IR spectra (middle) and close-up (right) of the supernatant of the TAPA-PMDA-COF reactions obtained from the reaction observation experiment in Figure S5 after 1 h at 200 °C (left). While in the supernatant of the alcohol containing reaction mixture (green) imide species could be detected, the supernatant of the water reaction mixture (blue) contains protonated amine and deprotonated pyromellitic acid linker molecules.

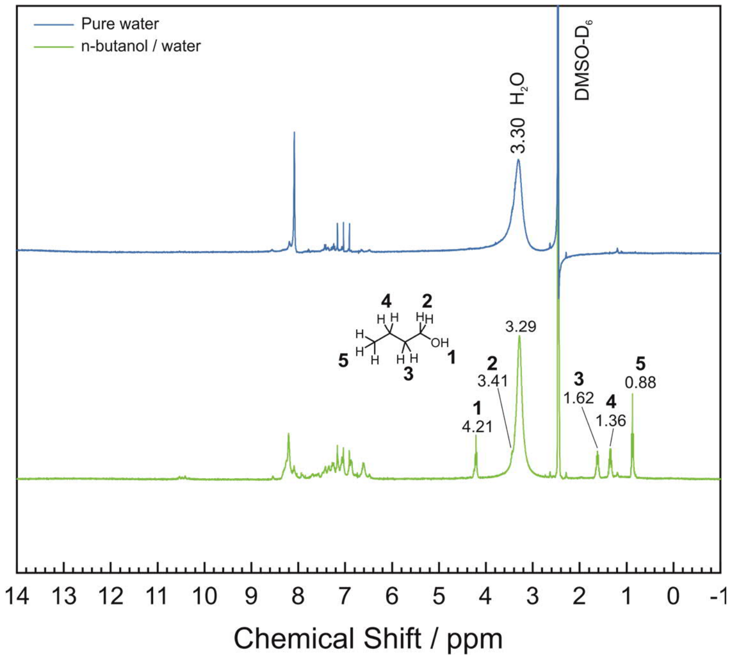

Figure S8. ^{1}H NMR spectra of the in the supernatant dissolved species of the TAPA-PMDA-COF reactions obtained from the reaction observation experiment in Figure S5 after 1 h at 200 °C revealing n-butanol residuals.

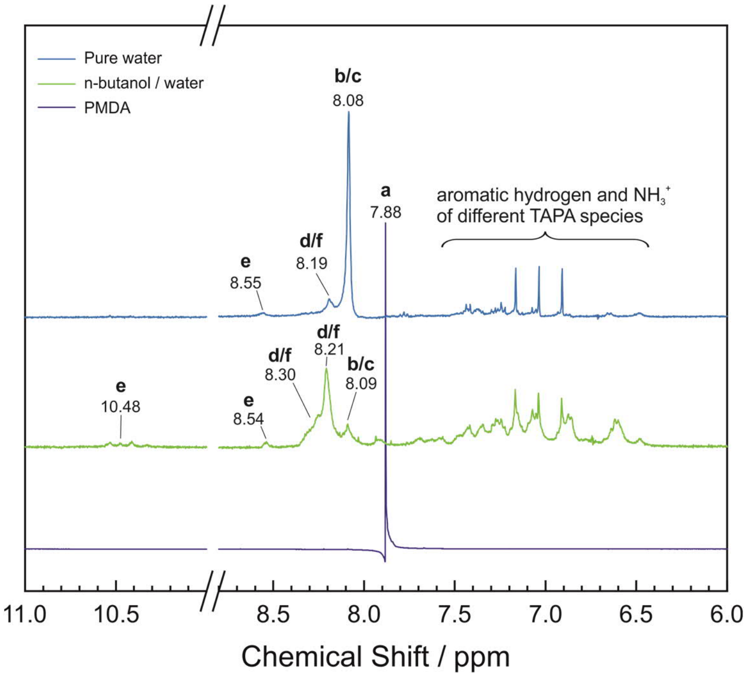

Figure S9. Close-up of the ^1H NMR spectra of the in the supernatant dissolved species of the TAPA-PMDA-COF reactions obtained from the reaction observation experiment in Figure S5 after 1 h at 200 °C together with the ^1H NMR spectrum of PMDA. While in the supernatant of the alcohol containing reaction mixture (green) imide (marked blue) and amic acid (marked red) species could be detected, the supernatant of the water reaction mixture (blue) contains predominantly deprotonated pyromellitic acid linker molecules (marked purple).

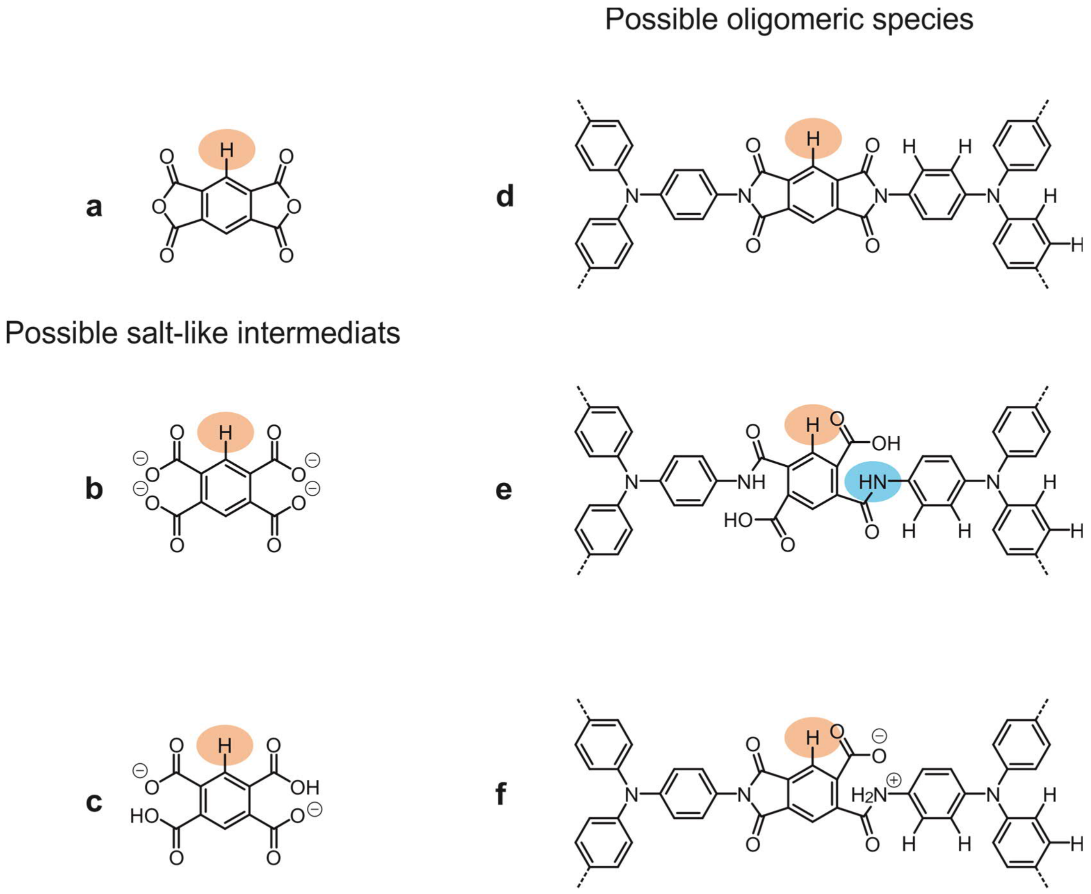

Figure S10. Chemical structures of species possibly present in the supernatant of the TAPA-PMDA-COF reactions obtained from the reaction observation experiment in Figure S5 after 1 h at 200 °C.

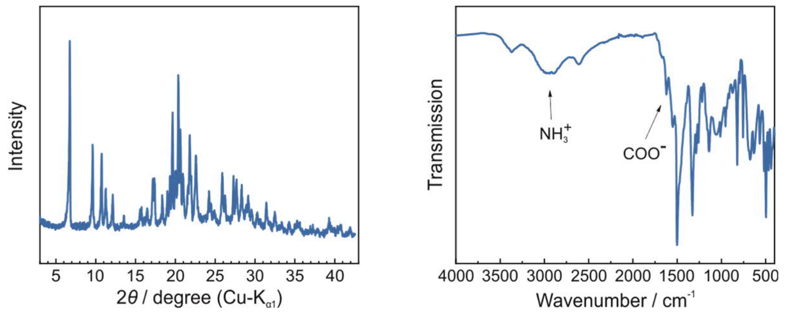

Figure S11. XRPD pattern (left) and FT-IR spectrum (right) of the isolated monomer-salt consisting of protonated TAPA and deprotonated pyromellitic acid linker molecules.

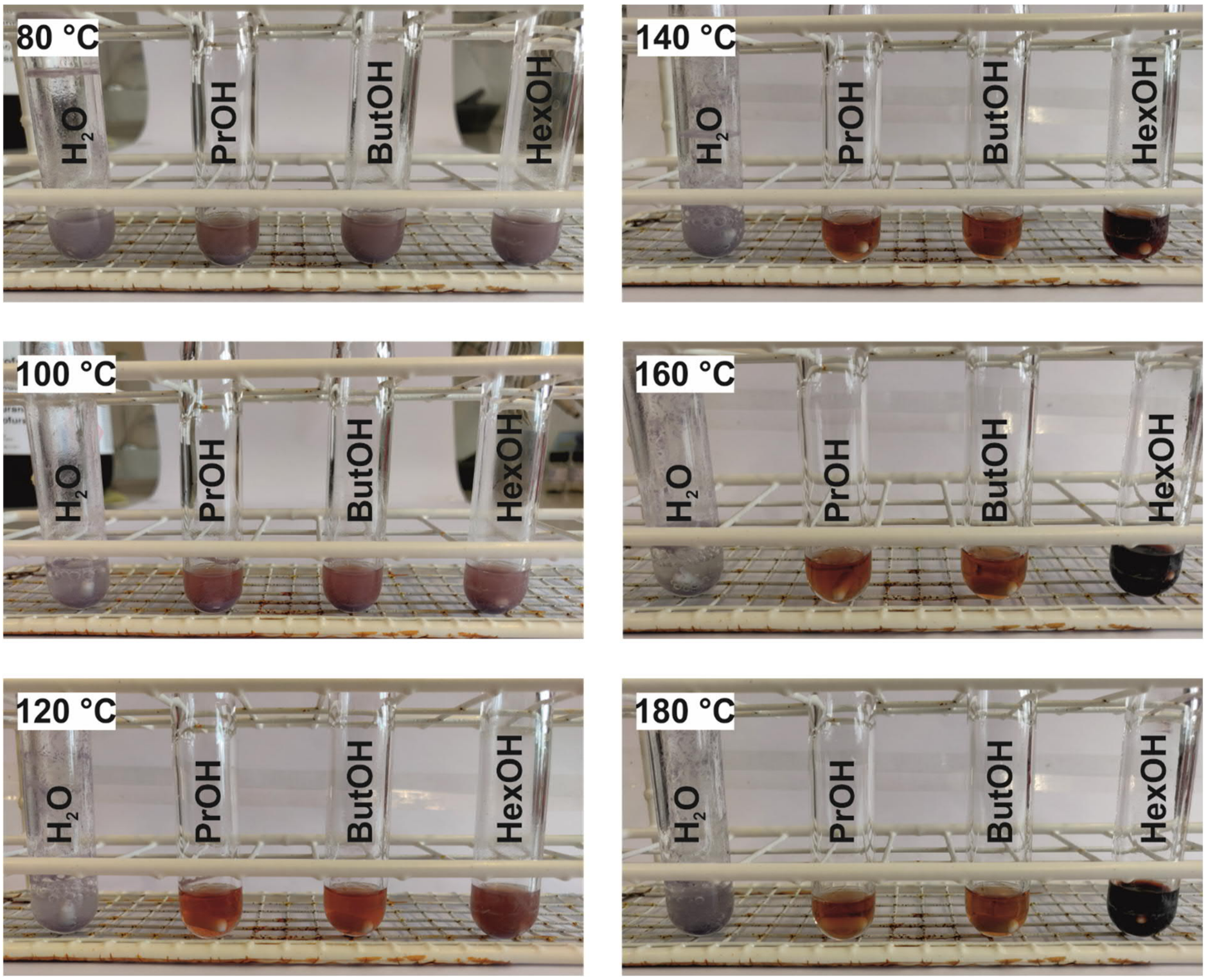

Figure S12. Solubility tests of TAPA in H_2O and different n-alcohols revealing poor solubility of TAPA in H_2O and good solubility in all the tested n-alcohols.

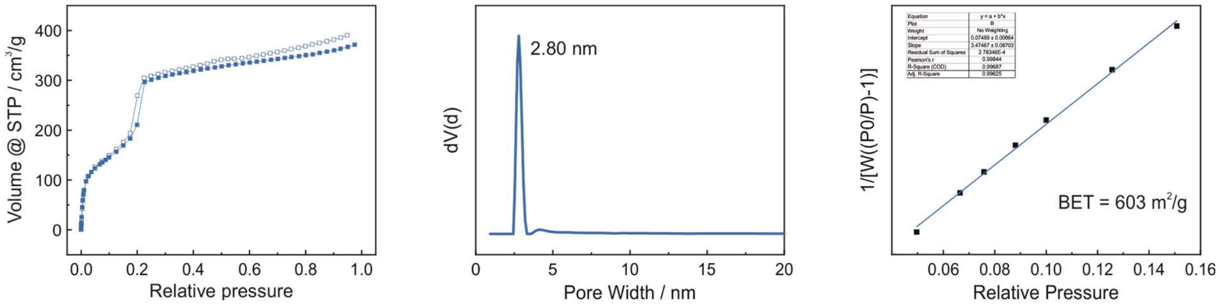

Figure S13. Ar sorption and desorption isotherm (left), calculated pore size distribution (center) and BET plot (right) of TAPA-PMDA-COF synthesized in 1.2 ml H2O/0.3 ml n-hexanol/0.04 ml pyridine at 180 °C for 24 h.

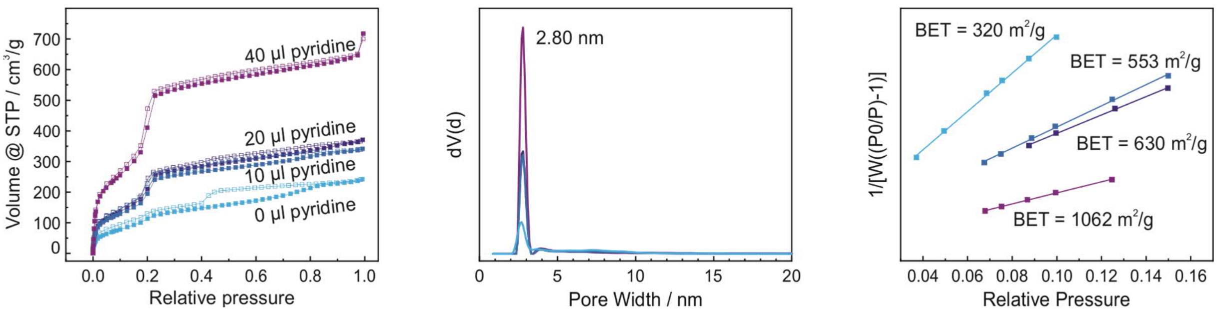

Figure S14. Ar sorption and desorption isotherms (left), calculated pore size distributions (center) and BET plots (right) of TAPA-PMDA-COF synthesized in 1.2 ml H_2O/0.3 ml n-hexanol with varying pyridine contents at 180 °C for 4 days.

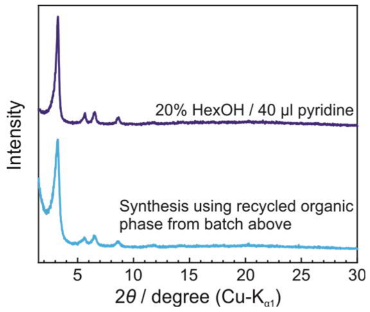

Figure S15. XRPD pattern of TAPA-PMDA-COF synthesized using fresh solvents (violet) and using the recycled organic phase (blue).

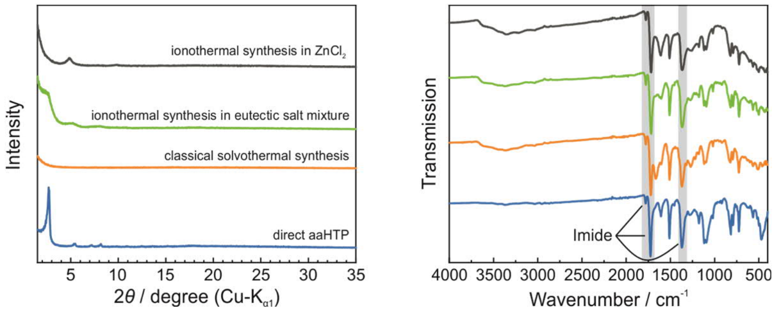

Figure S16. XRPD patterns (left) and FT-IR spectra (right) of the attempts to synthesize TAPE-PMDA-COF using different published synthetic procedures and the direct aaHTP.[4, 5]

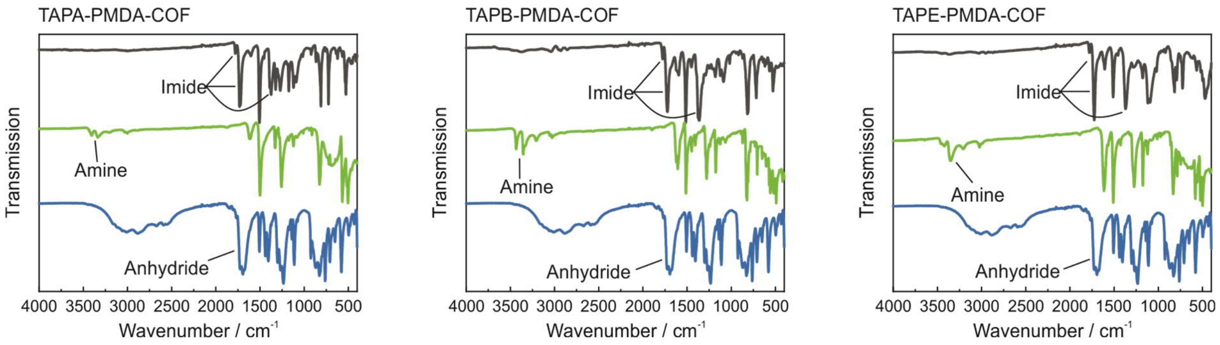

Figure S17. FT-IR spectra of TAPA-PMDA-, TAPB-PMDA- and TAPE-PMDA-COF (black) synthesized in optimized reaction conditions together with FT-IR spectra of their respective precursor molecules TAPA / TAPB / TAPE (green) and PMDA (blue). The absence of amine vibrational bands at 3367 cm^{-1} and anhydride vibrational bands at 1700 cm^{-1} in the COFs spectra indicate complete imide formation.

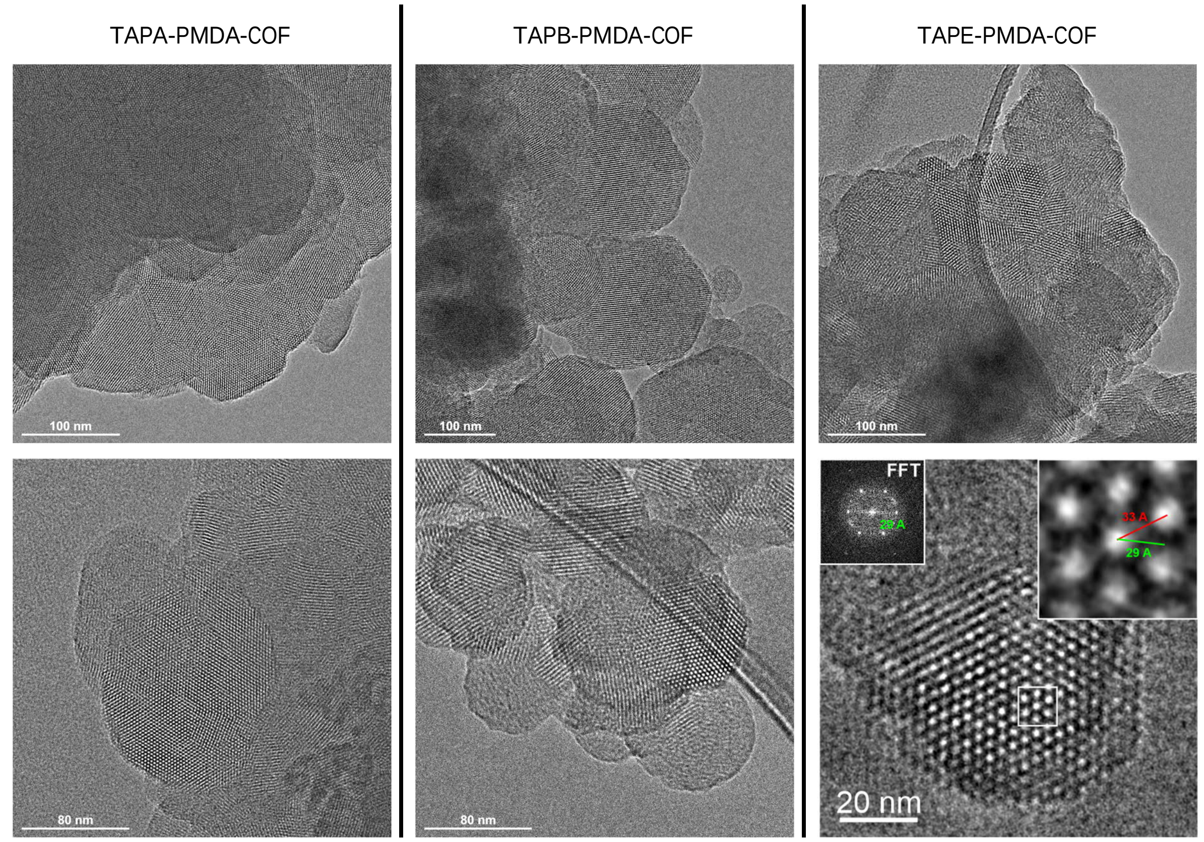

Figure S18. TEM images of TAPA-PMDA-COF (left), TAPB-PMDA-COF (center) and TAPE-PMDA-COF (right).

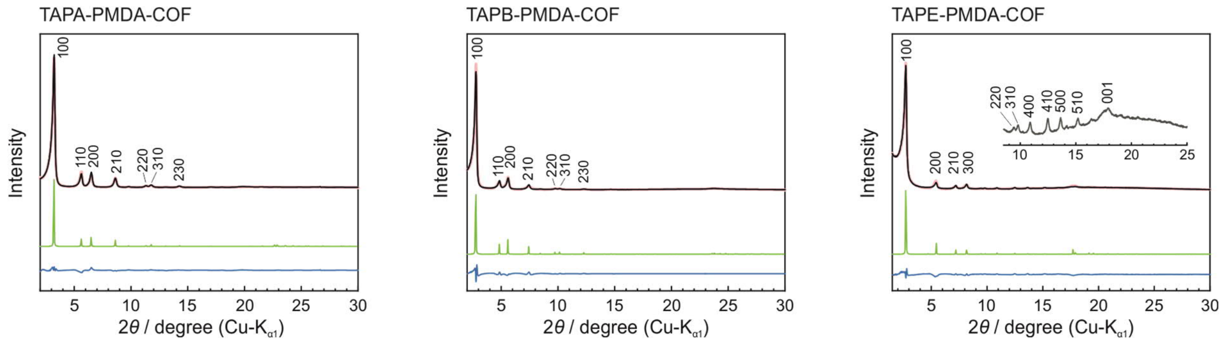

Figure S19. Experimental XRPD patterns of TAPA-PMDA-, TAPB-PMDA- and TAPE-PMDA-COF (black) together with the Rietveld fits (red), simulated patterns (green) and difference curves (blue). Simulations of the three COFs were performed based on the *P*622 space group for TAPA-PMDA-COF, $P\bar{3}1m$ space group for TAPB-PMDA-COF, and *P*622 space group for TAPE-PMDA-COF.

Table S1. Rietveld refinement of TAPA-PMDA-COF, TAPB-PMDA-COF and TAPE-PMDA-COF at room temperature.

Fitted Pattern	TAPA-PMDA-COF	TAPB-PMDA-COF	TAPE-PMDA-COF
Space group	*P*622	$P\bar{3}1m$	*P*622
Rwp (%)	7.63	9.37	5.47
Cell Volume (Å³)	3268	4225	6081
a (Å)	31.06(9)	36.0(4)	37.4(6)
b (Å)	31.06(9)	36.0(4)	37.4(6)
c (Å)	3.9(1)	3.75(6)	5.00(4)
**α** (°)	90	90	90
**β** (°)	90	90	90
**γ** (°)	120	120	120

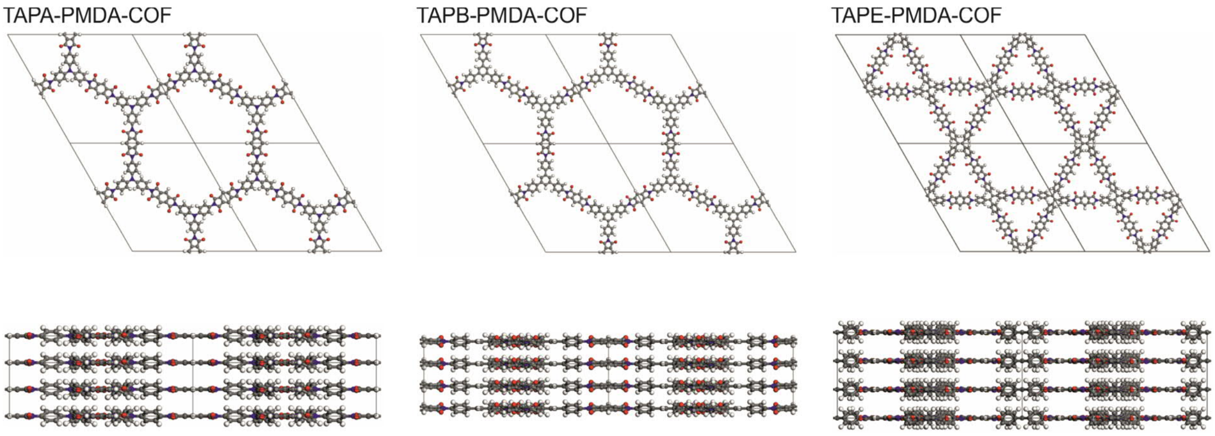

Figure S20. Simulated structures of the TAPA-PMDA-COF, TAPB-PMDA-COF and TAPE-PMDA-COF based on the space groups *P*622, $P\bar{3}1m$ and *P*622, respectively.

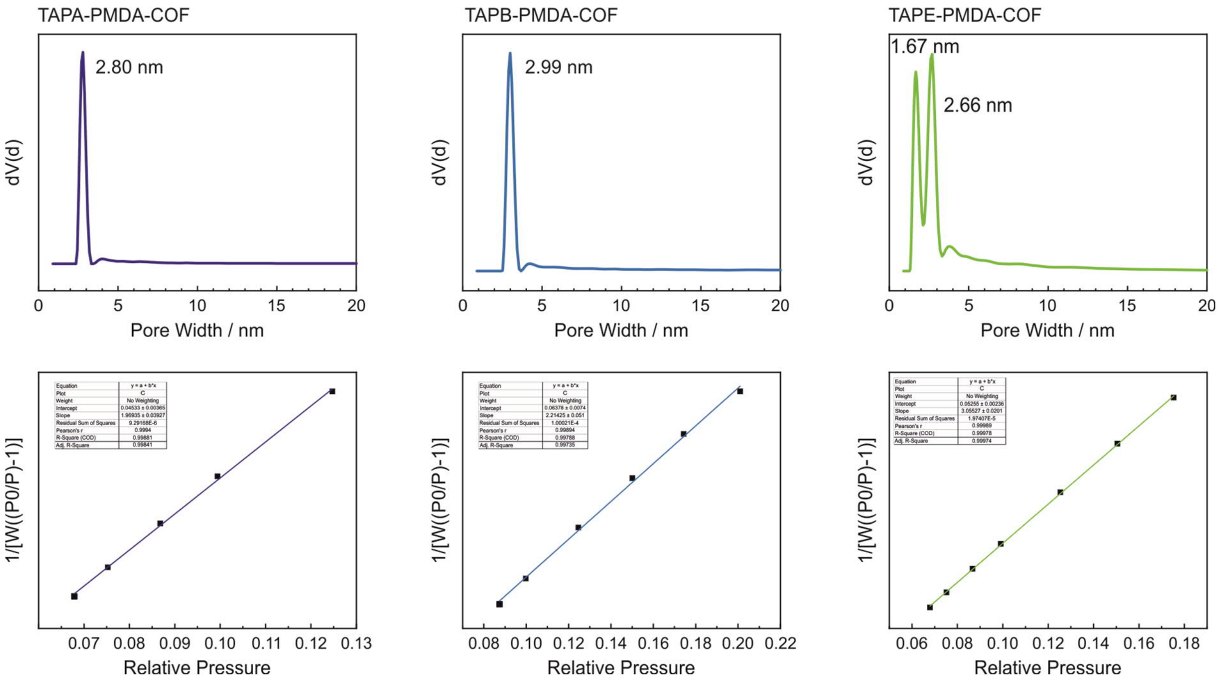

Figure S21. Calculated pore size distribution of TAPA-PMDA-COF (violet), TAPB-PMDA-COF (blue) and TAPE-PMDA-COF (green) together with their respective BET plots.

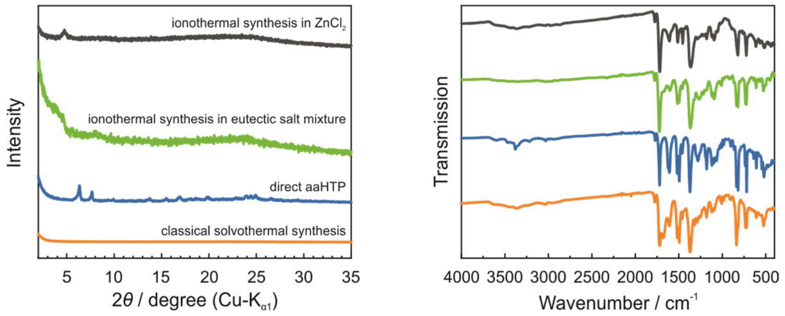

Figure S22. XRPD patterns (left) and FT-IR spectra (right) of the attempts to synthesize Py-imide COF directly from the precursor molecules using different published synthetic procedures.[4, 5]

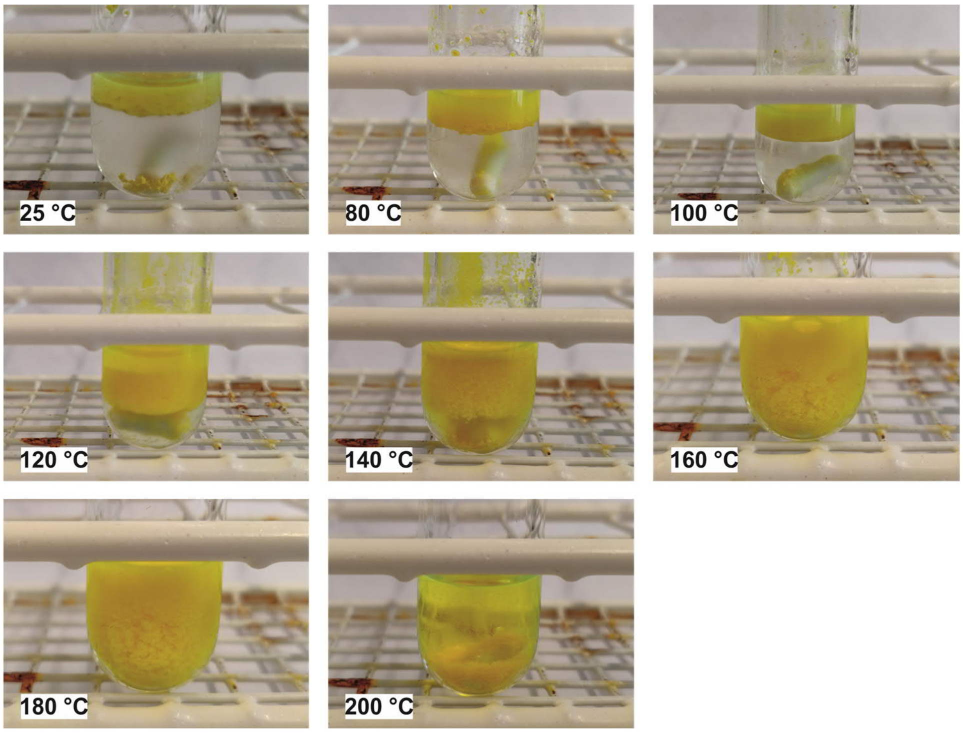

Figure S23. Solubility test of PyTTA in a mixture of 30 % n-butanol / H_2O revealing poor solubility of PyTTA up to 200 °C.

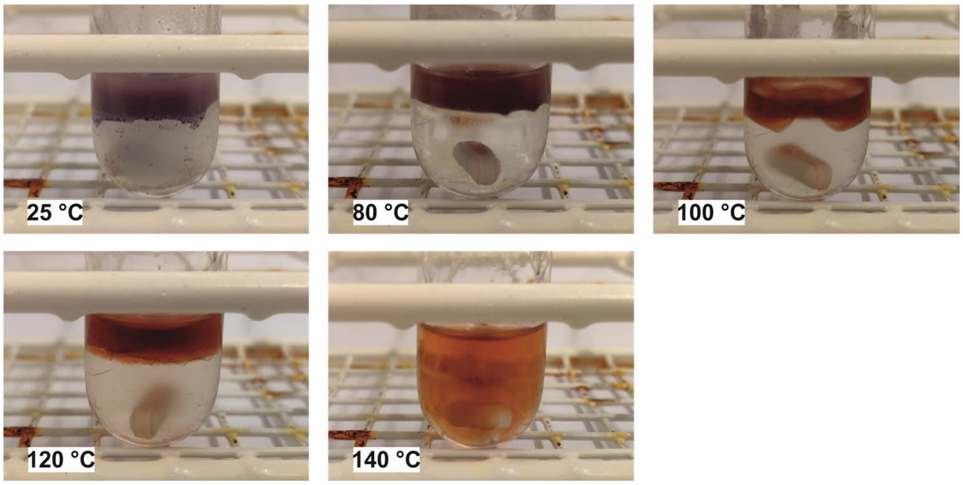

Figure S24. Solubility test of TAPA in a mixture of 30 % n-butanol / H_2O revealing complete dissolution of TAPA at 140 C.

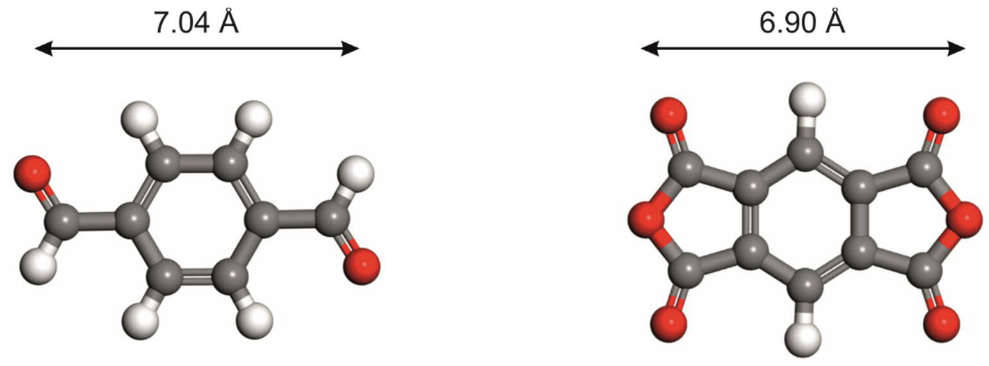

Figure S25. Calculated size of the linker molecules terephthaldehyde (left) and PMDA (right).

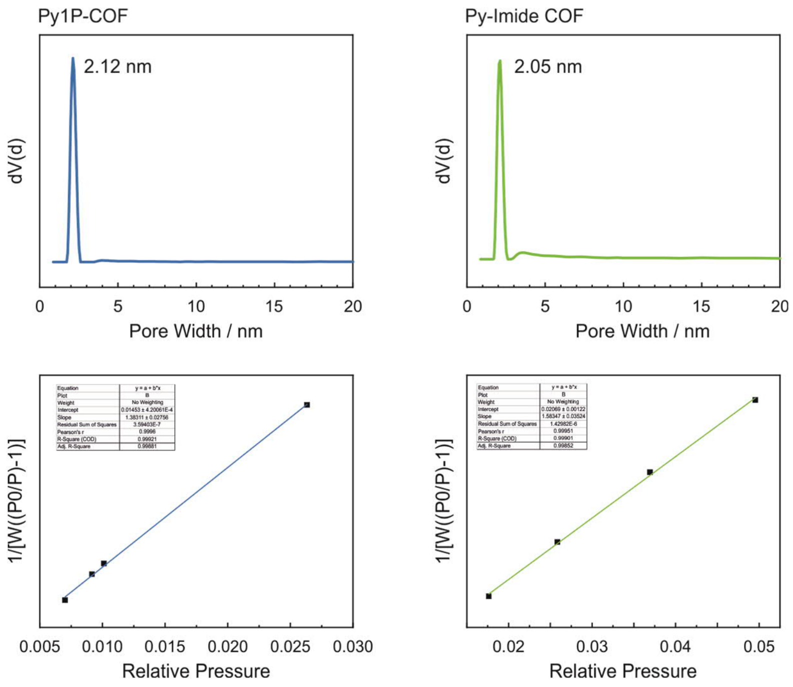

Figure S26. Calculated pore size distribution of Py1P-COF (blue) and Py-imide COF (green) together with their respective BET plots.

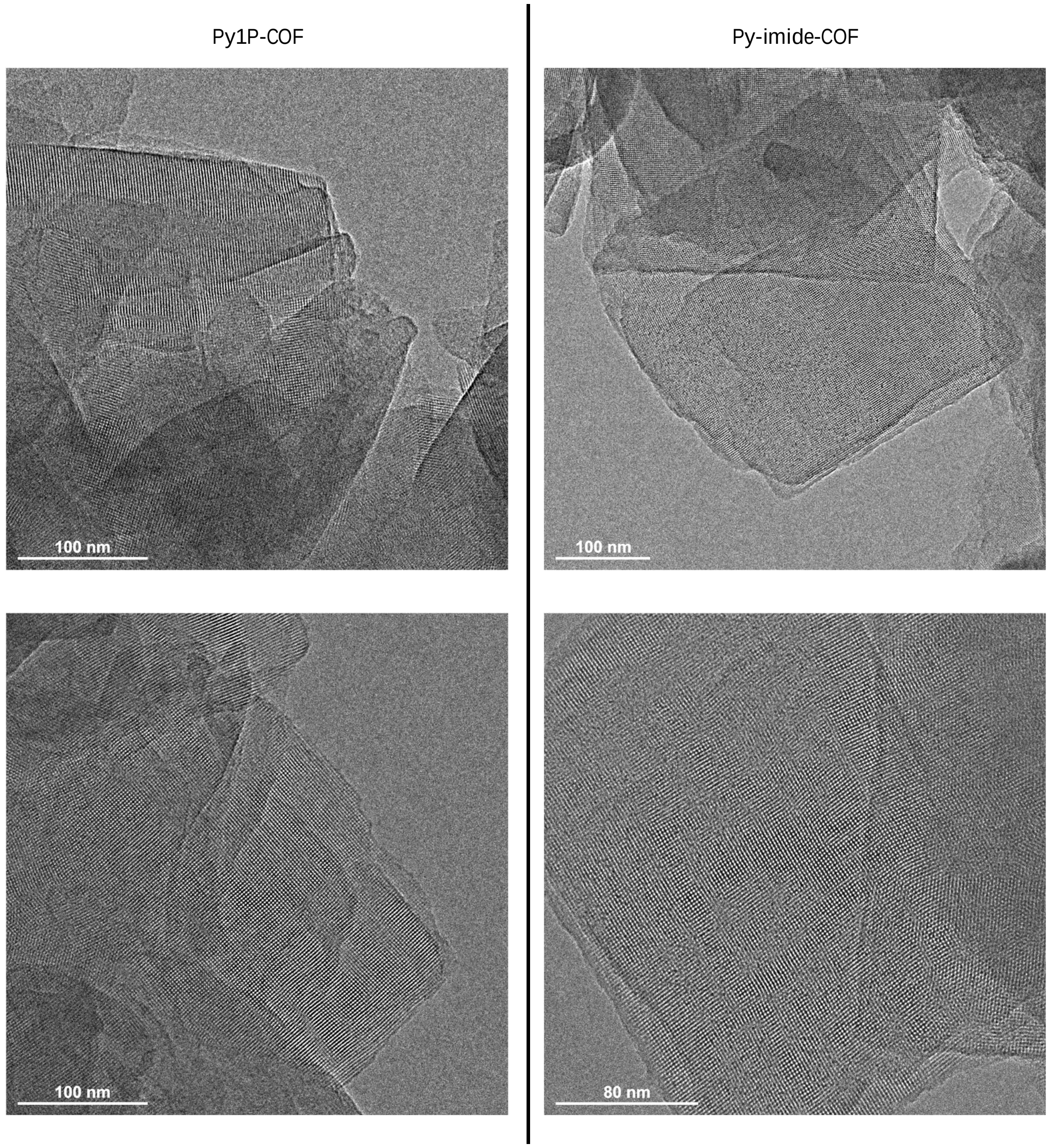

Figure S27. TEM images of Py1P-COF (left) and Py-imide COF (right).

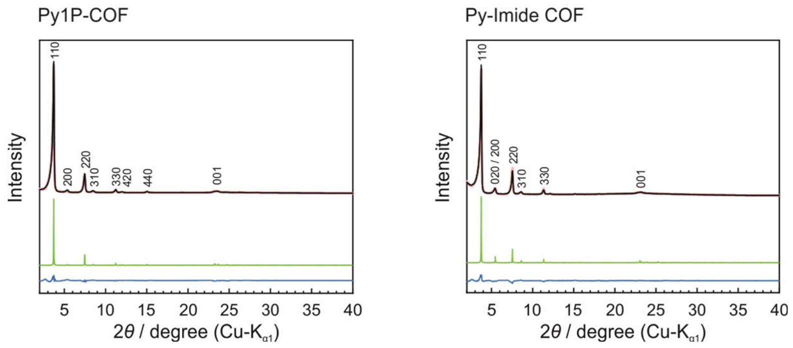

Figure S28. Experimental XRPD pattern of the imine-linked Py1P-COF and the imide-linked Py-imide COF (black) together with the Rietveld fits (red), simulated patterns (green) and difference curves (blue). Both COFs were simulated based on the *C*2/*m* space group.

Table S2. Rietveld refinement of Py1P-COF- and Py-imide COF at room temperature.

Fitted Pattern	Py1P-COF	Py-imide COF
Space group	*C*2/*m*	*C*2/*m*
Rwp (%)	7.4	5.3
Cell Volume (Å³)	4225	4252
a (Å)	38.0(1)	33.8(1)
b (Å)	32.5(1)	32.1(7)
c (Å)	3.82(3)	3.91(6)
**α** (°)	90	90
**β** (°)	63.4(2)	86.(8)
**γ** (°)	90	90

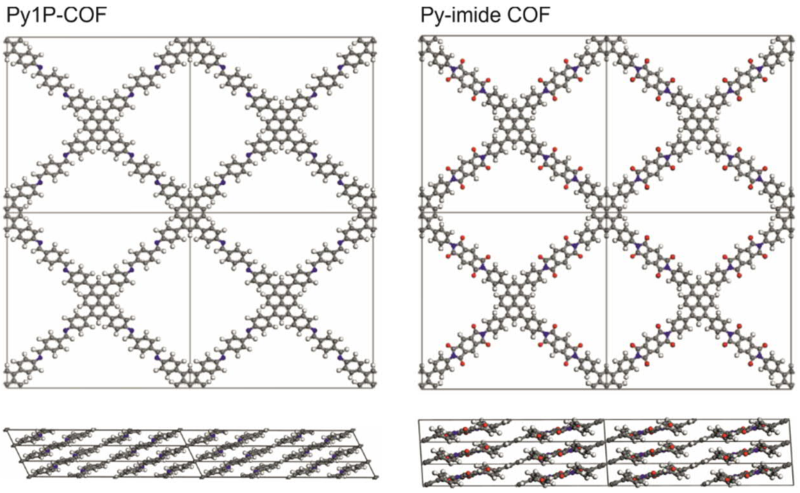

Figure S29. Simulated structures of the Py1P-COF and Py-imide COF based on the space group *C*/2*m*.

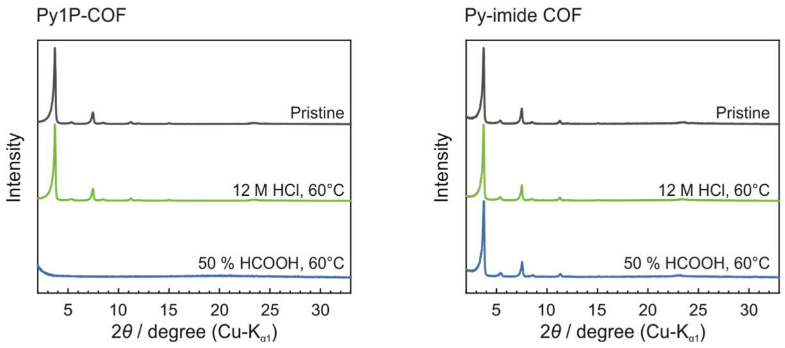

Figure S30. Comparison of the XRPD patterns of Py1P-COF and Py-imide COF pristine (black) and after treatment with concentrated hydrochloric acid (green) and formic acid (blue).

References

[1] Rietveld, H. M., A profile refinement method for nuclear and magnetic structures. 1969, *2* (2), 65-71.

[2] Coelho, A., Whole-profile structure solution from powder diffraction data using simulated annealing. *Journal of Applied Crystallography* 2000, *33* (3 Part 2), 899-908.

[3] D. R. Lide, *CRC Handbook of Chemistry and Physics*, 86 ed., Boca Raton : CRC Press, 2005.

[4] Q. Fang, Z. Zhuang, S. Gu, R. B. Kaspar, J. Zheng, J. Wang, S. Qiu, Y. Yan, *Nature communications* 2014, *5*;

[5] J. Maschita, T. Banerjee, G. Savasci, F. Haase, C. Ochsenfeld, B. V. Lotsch, 2020, *59*, 15750-15758.

Author Contributions

J. Maschita carried out the experiments. T. Banerjee and B. V. Lotsch supervised the project. The manuscript was written through contributions of all authors.
